# Investigation into Drug-Induced Liver Damage Using Multimodal Mass Spectrometry Imaging

**DOI:** 10.1021/jasms.4c00313

**Published:** 2025-01-17

**Authors:** Bryn Flinders, Lennart Huizing, Bhanu Singh, Heng-Keang Lim, Marjolein van Heerden, Filip Cuyckens, Ron M. A. Heeren, Rob J. Vreeken

**Affiliations:** † Maastricht MultiModal Molecular Imaging Institute (M4i), Division of Imaging Mass Spectrometry, Maastricht University, Universiteitssingel 50, 6229 ER Maastricht, The Netherlands; ‡ Janssen Research & Development, 1400 McKean Rd, Spring House, Pennsylvania 19477, United States; § Janssen Research & Development, Turnhoutseweg 30, 2340 Beerse, Belgium

## Abstract

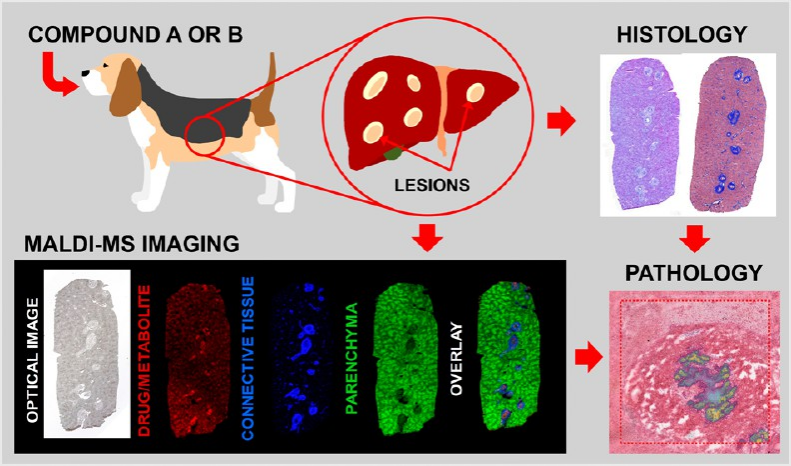

Drug toxicity during the development of candidate pharmaceuticals is the leading cause of discontinuation in preclinical drug discovery and development. Traditionally, the cause of the toxicity is often determined by histological examination, clinical pathology, and the detection of drugs and/or metabolites by liquid chromatography–mass spectrometry (LC-MS). While these techniques individually provide information on the pathological effects of the drug and the detection of metabolites, they cannot provide specific molecular spatial information without additional experiments. Matrix-assisted laser desorption/ionization-mass spectrometry imaging (MALDI-MSI) is a powerful, label-free technique for the simultaneous detection of pharmaceuticals, metabolites, and endogenous chemical species in tissue sections, which makes it suitable for mechanistic toxicological studies to directly correlate the distribution of the drug and its metabolites with histological findings. This capability was demonstrated by the analysis of the liver from dogs dosed with discontinued drug compound B and its N-desmethyl metabolite, compound A. Histological examination showed multifocal hepatocellular necrosis, bile duct hyperplasia, periportal fibrosis, and chronic inflammation. MALDI-MSI analysis of liver tissue dosed with only compound A indicated that liver lesions were associated exclusively with the parent compound, whereas liver lesions with compound B showed the presence of the parent compound and its two metabolites (compound A and an N-oxide metabolite). Using both positive and negative ion modes, simultaneous detection and identification of endogenous molecular markers of the connective tissue, blood vessels, liver parenchyma, and bile duct epithelium was achieved, allowing optimal visualization of histological lesions by mass spectrometry imaging.

## Introduction

The cost of developing a new pharmaceutical compound has increased steadily, primarily due to the high attrition rate in the clinical phase of drug development. This attrition significantly lowers the success rate of candidate pharmaceuticals reaching the market. The projected cost of successfully developing a pharmaceutical has reached $2.6 billion (USD) and continues to rise each year.^1^ A key factor contributing to this issue is the unexpected emergence of drug toxicity observed later in clinical trials, often leading to costly decisions to halt the further development of drug candidates. This problem is partly due to the limitations of existing preclinical animal models, which are not always effective in accurately assessing safety.^2^

During the preclinical phase of drug development, pharmaceutical industries typically employ techniques such as quantitative whole-body autoradiography (QWBA) to assess tissue distribution and the potential accumulation of drug-derived compounds. However, this technique is limited in that it cannot distinguish whether the observed signal represents the drug or metabolite due to the use of radiolabeled compounds.^3^ Subsequent drug metabolism and pharmacokinetic (DMPK) studies using liquid chromatography–mass spectrometry (LC-MS) can determine the presence and quantity of the drug and its metabolites in various tissues. However, all spatial information is lost due to homogenization of the tissues before analysis. Histopathological examination is a crucial part of toxicological investigations to evaluate safety in preclinical species and potentially study the underlying processes involved in the pathological findings. However, it lacks information about the contribution of the drug and its metabolites. The ability to simultaneously monitor the distribution of a drug, its metabolites, and endogenous chemical species and then correlate this information with standard histological staining would be a distinct advantage in pharmaceutical research and toxicological investigations.

Matrix-assisted laser desorption/ionization-mass spectrometry imaging (MALDI-MSI) is a label-free technique that has extensively been used to monitor the distribution of pharmaceutical compounds in a wide range of tissues.^4,5^ This technique can simultaneously monitor the distribution of the administered compound, its metabolites, and endogenous species, making it a valuable complementary tool for use in toxicological studies.^6,7^ Several studies have reported using this technology to investigate drug-induced tissue damage from pharmaceutical compounds. For example, MALDI-MSI was utilized to investigate the damage to rat kidneys following the administration of two novel prostaglandin E synthase 1 inhibitors. Using a combination of MALDI-MSI, LC-MS, and nuclear magnetic resonance (NMR), the crystalline deposits in areas associated with tissue damage were found to be composed of bisulphonamide. This example demonstrated the wealth of information that can be obtained from mass spectrometry imaging (MSI) in combination with other techniques, in comparison to relying solely on histological examination.^8^ Similarly, a combination of MALDI-MSI and desorption electrospray ionization-mass spectrometry imaging (DESI-MSI) was used to investigate crystal-like structures in formalin-fixed paraffin-embedded (FFPE) rabbit kidney tissue sections. The formation of these crystals was attributed to the demethylated/oxidized metabolite from a novel c-Met tyrosine kinase inhibitor. Other metabolites, including the demethylated, oxidized, and double oxidized variants, were also associated with the crystals, with minimal contribution from the parent drug.^9^

MALDI-MSI has also been used to investigate the mechanism of liver toxicity after administration of nevirapine, a non-nucleoside inhibitor of the HIV polymerase enzyme, to Norway rats.^10^ Distribution studies using MALDI-MSI localized nevirapine to the centrilobular region of the liver and identified colocalization of a sphingomyelin species in fibrotic liver tissue. In another study, the composition of renal deposits in rat kidneys was analyzed following oral administration of dabrafenib, an ATP-competitive inhibitor of RAF kinase activity, using a combination of MALDI-MSI and laser desorption ionization-mass spectrometry imaging (LDI-MSI). MALDI-MSI showed that the metabolite was localized around the renal deposits, while LDI-MSI revealed that the renal deposits were primarily composed of calcium phosphate. The hypothesized cause for the renal toxicity and deposits is that age-related responses to the drug affected younger rats more severely than older rats.^11^

Recently, MALDI-MSI was used to investigate renal toxicity in rats treated with sotorasib, a KRAS^G12C^ covalent inhibitor used for the treatment of tumors with this specific mutation. The study revealed degeneration and necrosis of the proximal tubular epithelium in the outer stripe of the outer medulla. The metabolites generated via the mercapturate pathways were hypothesized to be the cause of the toxicity, based on the localization of these thiol mercapturic acid metabolites in the affected areas, observed using a combination of histology and MALDI-MSI.^12^

The work reported here aims to use a multimodal imaging approach to investigate whether the toxicological findings of the multifocal hepatocellular necrosis and bile duct hyperplasia, along with periportal fibrosis and chronic inflammation, observed in livers of dogs dosed with two candidate pharmaceutical compounds can be linked to either the dosed compounds or its related metabolite(s). Additionally, the data revealed the synergistic potential for identifying off-target molecular markers of bile-duct epithelium health.

## Methods

### Animal Dosing

Two non-GLP, 14-day repeat dose, investigative oral toxicity studies were conducted using compound A or B in Beagle dogs. Compounds A and B were formulated in 20% hydroxypropyl-β-cyclodextrin and administered by the oral route (gavage) to male dogs (n = 3 for compound A and n = 2 for compound B) at 65 mg/kg for 2 weeks. The dogs were approximately 10–12 months old at the start of dosing. Dogs were observed for clinical signs at 2 and 6 h postdose on weekdays and at 2 h postdose on weekends. Food consumption was assessed pretreatment and daily for the remainder of the study. Blood samples for toxicokinetics, hematology, and clinical chemistry assessments were collected into K_2_ EDTA tubes on day 0 and at 1, 2, 5, 7, and 24 h postdose from the jugular vein. Plasma was harvested by centrifugation at 3500 rpm and 5 °C for 10 min. At the end of the study, all dogs were fasted overnight, euthanized, and necropsied on day 14. The thoracic and abdominal cavities were examined, and major tissues, including the liver with gross lesions, were collected. Liver samples were taken, with a portion fixed in 10% neutral buffered formalin for histological analysis, and smaller sections, preferably those containing gross lesions, were snap-frozen in liquid nitrogen and processed for imaging mass spectrometry (described below). Control dog liver (Beagle dog, n = 1) was obtained from an ongoing study at Janssen Pharmaceutica N. V. The local animal ethics committee approved the study, which was conducted in facilities accredited by national institutions adhering to AAALAC guidelines.

### Materials

2,5-Dihydroxybenzoic acid (DHB; 98%), α-cyano-4-hydroxycinnamic acid (CHCA; ≥ 98%), 9-aminoacridine hydrochloride monohydrate (9AA; 98%), norharmane (NOR; crystalline), potassium chloride (KCl; ≥ 99.0%), sodium chloride (NaCl; ≥ 99.5%), sodium taurodeoxycholate (TDCA; ≥ 95%), sodium taurocholate hydrate (TCA; ≥ 97.0%), and trifluoroacetic acid (TFA; ≥ 99.0%) were purchased from Sigma-Aldrich (Zwijndrecht, The Netherlands). ULC/MS-CC/SFC–grade methanol (MeOH), water (H_2_O), and HPLC-grade chloroform (CHCl_3_) were purchased from Biosolve (Valkenswaard, The Netherlands). Compounds A and B (1 mg/mL in MeOH, diluted to 100 μg/mL in 70% MeOH) were kindly provided by Janssen Research & Development (Beerse, Belgium). A mixture of sulfatides (from porcine brain) and reference phospholipid standards SM (d18:1/16:0), PC (16:0/16:0), PC (18:0–20:4), and PI (18:0–20:4) were purchased from Avanti Polar Lipids (Alabaster, AL, USA) at >99% purity (the stock lipid standards were diluted to a concentration of 1 mg/mL in 2:1 v/v CHCl_3_: MeOH). To prepare the sodiated and potassiated bile acid standards, a 1 mg/mL bile acid solution in 50% MeOH was mixed with a 10 mg/mL solution of NaCl or KCl in 50% MeOH in a 1:1 (v/v) ratio. Subsequently, 1 μL of this mixture was applied to a clean indium tin oxide (ITO) glass slide (4–8 Ω resistance, Delta Technologies, Loveland, CO, USA) and allowed to dry. After drying, 1 μL of the matrix solution (15 mg/mL DHB in 2:1 v/v CHCl_3_: MeOH with 0.2% TFA) was spotted on top and left to dry.

### Tissue Preparation

Liver tissue obtained from control and dosed animals was sectioned using a Microm HM535 cryomicrotome (Microm International, Walldorf, Germany) at −20 °C to produce 10 μm thick sections, which were thaw-mounted onto clean ITO-coated glass slides. Consecutive tissue sections were thaw-mounted on Superfrost Plus microscope glass slides (VWR International, Leuven, Belgium) for histological comparison. Tissue sections were optically scanned before matrix application using a Nikon Super CoolScan 5000 ED (Nikon Corporation, Tokyo, Japan) with VueScan 9.7.51 software (Hamrick Software; www.hamrick.com) to produce high-quality images (4000 dpi) for coregistration with the MALDI-MS images.

### Matrix Application

Tissue sections were coated with 15 mg/mL of DHB in CHCl_3_:MeOH (2:1, v/v) with 0.2% TFA using the SunCollect automated pneumatic sprayer equipped with a dispenser system (Sunchrom GmbH, Friedrichsdorf, Germany) in a series of 15 layers. The initial seeding layer was applied at 10 μL/min and subsequent layers were applied at 20, 30, and 40 μL/min (speed: 450 mm/min, track spacing: 2 mm, height: 23 mm). Additionally, some tissue sections were coated with 10 mg/mL 9AA in MeOH: H_2_O (70:30, v/v) in a series of 10 layers. The initial seeding layer was performed at 10 μL/min, followed by subsequent layers at 15, 20, and 25 μL/min (speed: 450 mm/min; track spacing: 2 mm; height: 25 mm). The nitrogen gas pressure was set to 2 bar. For high spatial resolution, consecutive tissue sections were coated with norharmane using the HTX sublimator (HTX Technologies, Chapel Hill, NC, USA) at a temperature of 140 °C for 200 min.

### Instrumentation

#### MALDI-MS Imaging

High-speed imaging was performed on a Bruker RapifleX MALDI Tissuetyper system (Bruker Daltonik GmbH, Bremen, Germany). A more detailed description of this instrument has previously been reported.^13^ The instrument was operated in reflectron mode in both positive and negative ion modes in the mass range *m*/*z* 400–1000. The laser power was 60%, the number of laser shots was 100, and the laser frequency was 10 kHz. The instrument was calibrated before analysis using red phosphorus clusters in both polarities.^14^ Images of the whole tissue sections were acquired using a 50 × 50 μm raster (25 × 25 μm beam scan area) for the larger tissue sections and a 30 × 30 μm raster (15 × 15 μm beam scan area) for the smaller tissue sections. Subsequently, areas of consecutive tissue sections were selected and imaged using a 10 × 10 μm raster (5 × 5 μm beam scan area). The images were generated using FlexImaging 5.0 software (Bruker Daltonik GmbH) and normalized to the total ion current (TIC).

High spatial resolution imaging (5 × 5 μm) was performed using a Bruker timsTOF flex (Bruker Daltonik GmbH). The instrument was operated in negative ion mode in the mass range *m*/*z* 400–1000. The laser power was 50%, the number of laser shots was 50, and the laser frequency was 1000 Hz. The instrument was calibrated before analysis using red phosphorus clusters, and online calibration was maintained throughout the measurements using the theoretical masses for [TCA-H]^−^ at *m*/*z* 514.2844 and [PI (38:4)-H]^−^ at *m*/*z* 885.5499. The images were then generated using SCiLS lab software 2024a (Bruker Daltonik GmbH).

#### MALDI-FTICR-MS Imaging

To confirm the chemical composition, high-mass resolution measurements (100,000 at *m*/*z* 500) were performed on a Bruker SolariX Fourier transform ion cyclotron resonance (FTICR) mass spectrometer (Bruker Daltonik GmbH) equipped with a 9.4 T superconducting magnet. The instrument was operated in both positive and negative ion modes in the mass range *m*/*z* 100–2000. The instrument was calibrated before analysis using red phosphorus clusters in both polarities. The laser power was 30% (2 kHz) with an accumulation of 100 shots and a “minimum” laser focus. Images were acquired with a pixel size of 50 μm. A 95% data-reduced profile spectrum and spectrum peak list was saved for further analysis. The images were generated by using FlexImaging 4.1 software (Bruker Daltonik GmbH) and were normalized to the total ion current (TIC).

#### MALDI-MS/MS

Further confirmatory analysis was conducted using high-mass resolution tandem mass spectrometry (MS/MS) using a Q Exactive Plus (Thermo Fisher Scientific GmbH, Bremen, Germany) coupled to a reduced pressure ESI/MALDI ion source (Spectroglyph LLC, Kennewick, WA, USA). A more detailed description of the ion source is described elsewhere.^15^ The instrument was operated in both positive and negative ion modes with a mass resolution of 120,000 (fwhm at *m*/*z* 400) with a laser pulse energy of 1.6 μJ (1 kHz). Using the ESI source, the instrument was calibrated prior to analysis with Pierce LTQ Velos positive and negative ion mode calibration solutions (Thermo Fisher Scientific GmbH, Bremen, Germany). The previously identified lipids associated with different liver anatomical features (e.g., blood vessels, bile ducts, connective tissue, parenchyma, and bile epithelium) served as a guide for analysis.^16^ An initial MS spectrum was acquired, after which tandem mass spectrometry was performed on selected masses using an isolation window of ±0.7 Da with an injection time of 2000 ms. The MS and MS/MS spectra were processed by using Xcaliber software. The spectra were then exported as a text file and imported into mMass v 5.5.0 software for postprocessing and visualization purposes.^17^ The generated MS and MS/MS spectra from the tissue sections were manually compared to those of commercial standards, literature sources, and databases such as Lipid Maps (www.lipidmaps.org) and the ALEX123 lipid calculator (www.alex123.info). The product ions observed in each MS/MS spectrum for the selected molecular species were annotated based on literature and the recently proposed nomenclature for lipids.^18^

### Histological Staining

Frozen liver tissue slices, which were consecutive tissue sections to those used for mass spectrometry imaging (10 μm thick), were stained with hematoxylin and eosin (H&E) and Masson’s trichrome (MTC) according to standard internal procedures. Postimaging H&E staining was performed on the samples immediately after analysis or following matrix removal by immersion in 100% EtOH for 30 s. The tissue sections were then sequentially washed with 70% EtOH (two washes) and deionized water for 2 min each. Next, the tissue sections were stained with hematoxylin for 3 min and subsequently washed with running tap water for 3 min. The tissue sections were then stained with eosin for 30 s and washed with running tap water for 1 min. After staining, the samples were placed into 100% EtOH for 1 min, followed by xylene for 30 s. Once dried, glass coverslips were placed onto the stained tissue sections using Entellan mounting medium. All stained tissue sections were then scanned using a Leica Aperio CS2 digital pathology slide scanner (Leica Biosystems, Wetzlar, Germany).

## Results and Discussion

### Histological Examination

**Figure 1:**
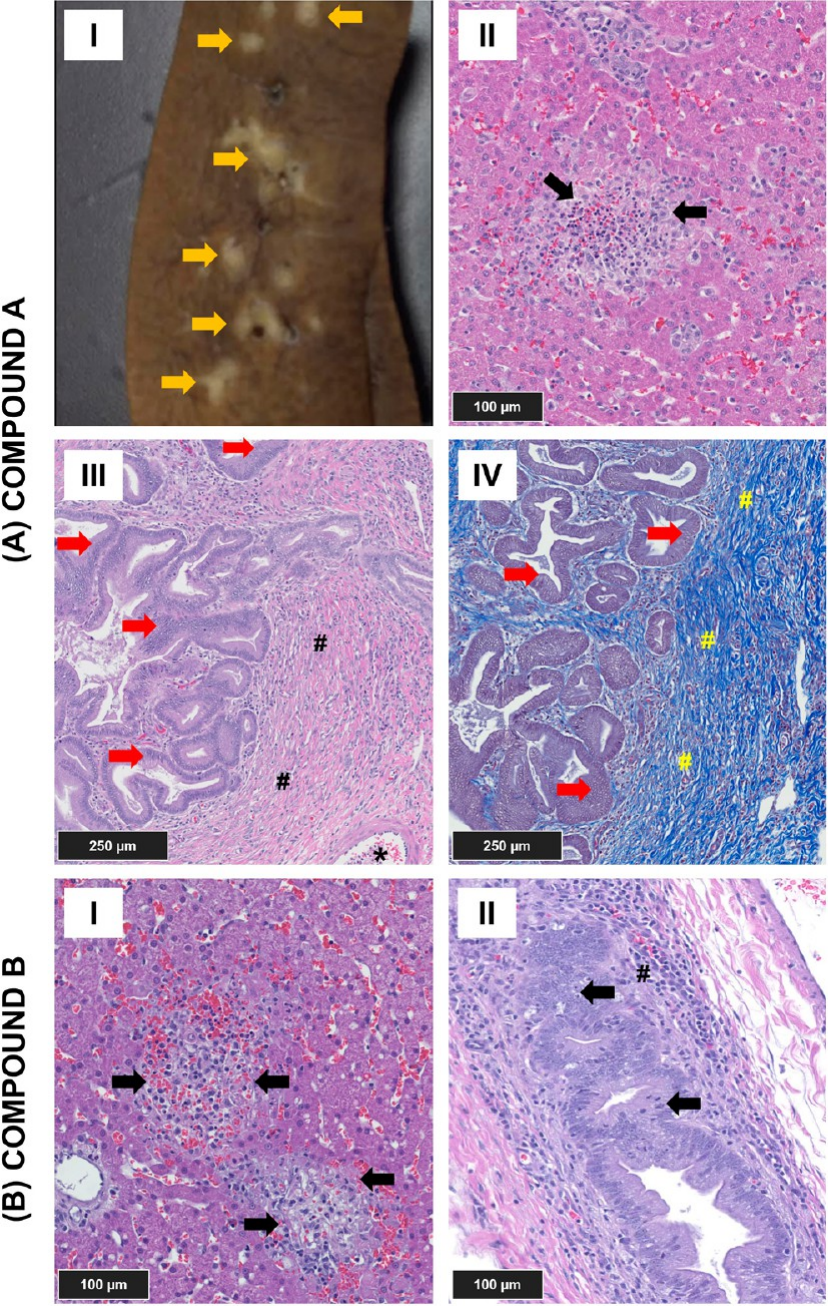
Pathological features of dog liver treated with compounds A and B (65 mg/kg/day). (A) Compound A–dosed liver showing (I) a gross image displaying multifocal areas of white discoloration (orange arrows) in the hepatic parenchyma, (II) H&E stain revealing a focus of hepatocellular necrosis (black arrows), with hemorrhage and mixed cell infiltrate, mainly macrophages, lymphocytes, and a few neutrophils, (III) H&E stain of the portal area, showing bile duct hyperplasia (red arrows) with peri-ductal fibrosis (#) and an adjacent blood vessel (*), and (IV) Masson’s trichrome stain of the portal area with bile duct hyperplasia (red arrows) and blue staining of connective tissue, consistent with collagen and periductal fibrosis (#). (B) Compound B–dosed liver showing (I) H&E stain of the dog liver, revealing foci of hepatocellular necrosis (black arrows), with hemorrhage and mixed cell infiltrate, (II) H&E stain of the portal area showing bile duct (black arrows) degeneration and regeneration (mitotic figures and hyperplasia), and peri-ductal mixed cell infiltrate (#).

The liver was identified as a target organ for both compounds, showing increases in the levels of liver enzymes (ALT, ALP, and GGT). The tissues treated with compound A (Figure 1A, I–IV) and compound B (Figure 1B, I–II) exhibited similar morphological changes. Both livers exhibited minimal to moderate hepatocellular necrosis, characterized by randomly distributed multifocal areas of necrotic hepatocytes intermixed with a few neutrophils. Minimal degradation of the bile duct epithelium was observed in medium- and large-sized bile ducts. This degeneration was associated with regenerative changes, including mitosis of bile duct epithelium and hyperplasia of bile duct and oval cells and infiltration of granulocytes in the portal areas. Microscopic changes in the portal areas were more pronounced for compound A compared to those seen with compound B. The livers of dogs treated with compound A displayed noticeable gross findings, including multifocal white discoloration, which correlated microscopically with bile duct hyperplasia surrounded by moderate portal tract fibrosis, often extending into adjacent periportal areas.

### Standard Analysis and On-Tissue Matrix Optimization

Pure standards of compounds A and B (100 μg/mL in 70% MeOH) were analyzed to obtain mass spectral reference profiles. The MALDI-MS spectrum of compound A exhibited a strong peak at *m*/*z* 502.15 (Figure S1A), while the MALDI-MS spectrum of compound B displayed a strong peak at *m*/*z* 516.17 (Figure S1B). The analysis indicated that both species were detected as only protonated ([M + H]^+^) species. Interestingly, no sodium or potassium adducts were observed for either compound. MALDI-MS/MS was performed to determine the product ions of both compounds for later confirmation. For proprietary reasons, the spectra cannot be shown.

Matrix evaluation tests were conducted using common small molecule positive ion matrices on tissue samples dosed with compound A. The initial tests were performed with 5 mg/mL CHCA in 70% ACN with 0.2% TFA and 15 mg/mL DHB in 70% MeOH with 0.2% TFA. The optical image (Figure S2A, I) shows the matrix application appeared to be homogeneous across all the tested matrices. Figure S2A, II shows the distribution of compound A with all the tested matrices. The images indicate that compound A could not be detected with CHCA. In contrast, compound A could be detected with DHB, but extensive delocalization was observed. Despite attempts to optimize the sprayer settings, this delocalization could not be avoided. However, the intensities of the molecular species that define the connective tissue and parenchyma were low with these matrices. As one of the goals is to simultaneously detect the drugs, metabolites, and endogenous species, the addition of chloroform to the matrix was then investigated (15 mg/mL DHB in 1:1 (v/v) CHCl_3_:MeOH with 0.2% TFA). The image indicates the presence of the drug, but this time no delocalization was observed. It also shows the drug was localized in the lesions, and the addition of chloroform improved the simultaneous detection of endogenous species. Further addition of chloroform (15 mg/mL in 2:1 (v/v) CHCl_3_:MeOH with 0.2% TFA) again showed the presence of the drug in the lesions, with an increased signal observed in both the connective tissue (Figure S2A, III) and parenchyma (Figure S2A, IV). Additionally, the distribution of the endogenous species was more clearly defined, as demonstrated in the overlay (Figure S2A, V). Based on these findings, this matrix was selected as the optimal choice for the detection of the compound, its metabolites, and endogenous species. These improvements are also highlighted in the graph shown in Figure S2B, which depicts the average intensity of each of the selected masses within the tissue boundaries.

### Multimodal Imaging of Compound A Dosed Liver Tissue Sections

**Figure 2:**
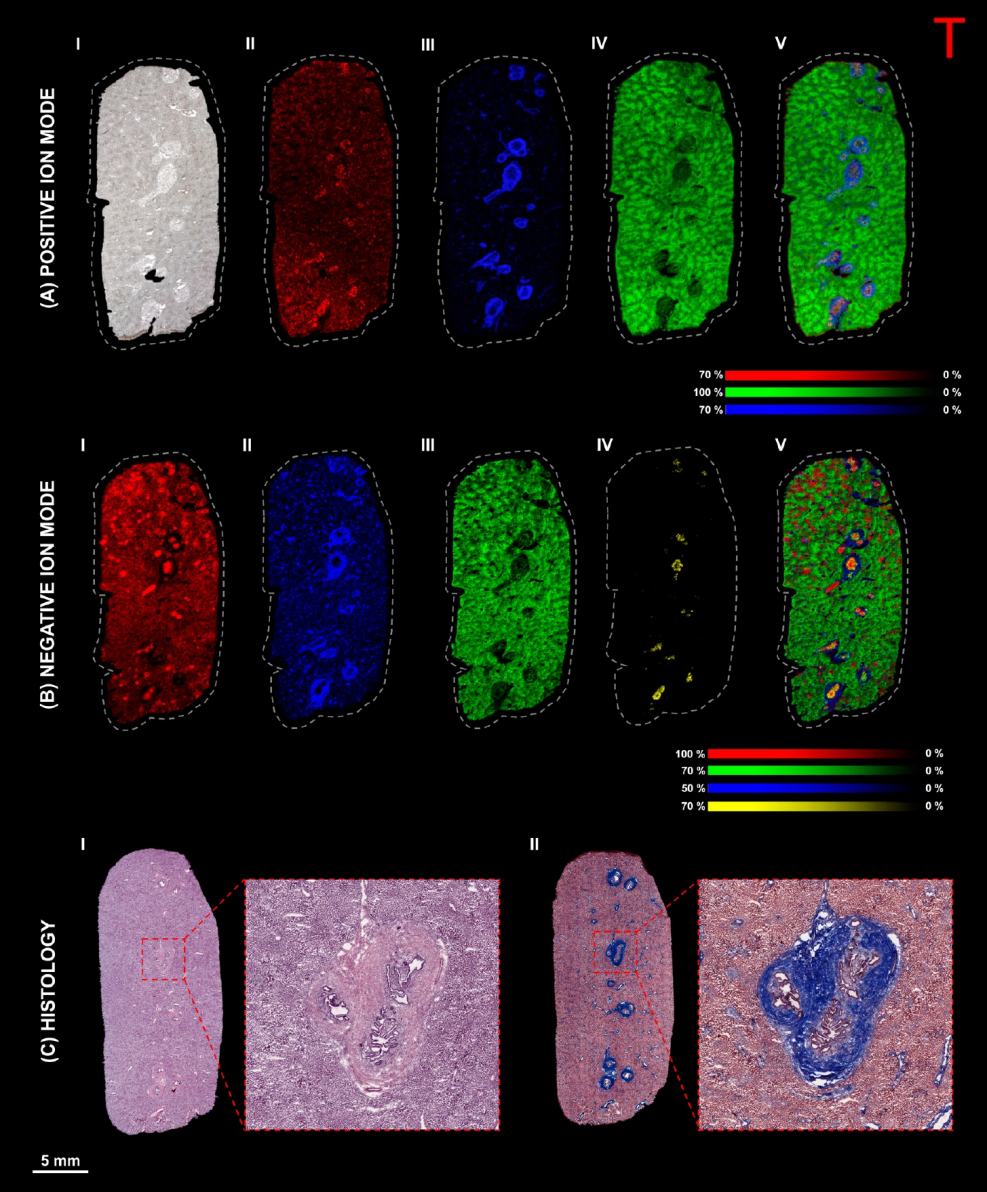
MALDI-MS imaging of compound A-dosed dog liver tissue (dog 3). (A) MALDI-MS imaging in positive ion mode showing (I) an optical image of the tissue section before matrix application. MALDI-MS images showing the distribution of (II) compound A ([M + H]^+^) at *m*/*z* 502.15, (III) ([PC (16:0_16:0) + K]^+^) at *m*/*z* 772.52, (IV) ([PC (18:0_20:4) + K]^+^) at *m*/*z* 848.55, and (V) the overlay of selected species. (B) MALDI-MS imaging in negative ion mode showing the distribution of (I) taurocholic acid ([M – H]^−^) at *m*/*z* 514.28, (II) ([SM (18:1_16:0) – CH_3_]^−^) at *m*/*z* 687.54, (III) ([PI (18:0_20:4) – H]^−^) at *m*/*z* 885.55, (IV) ([ST–OH (18:1_24:0) – H]^−^) at *m*/*z* 906.64, and (V) the overlay of selected species (spatial resolution 50 × 50 μm, normalized with TIC). (C) Histology of consecutive sections stained with (I) H&E and (II) MTC protocols (insets show areas chosen for high spatial resolution imaging).

The dosed tissue sections were then analyzed following the optimization of the matrix solution. The MALDI-MS images of liver tissue sections dosed with compound A are shown in Figure 2.

Several lesions can be observed throughout the liver tissue section dosed with compound A (Figure 2A, I). The size of these lesions varied greatly, with diameters ranging from 0.5 to 2 mm. The distribution of compound A ([M + H]^+^) at *m*/*z* 502.15 (Figure 2A, II) appeared to be homogeneously distributed throughout the parenchyma, with patches of higher intensity in the center of the lesions. Only compound A could be detected as the putative toxic phenolic metabolite previously identified with LC-MS was not observed, likely due to low concentrations (data not shown). The identity of compound A was confirmed by MALDI-MS/MS and MALDI-FTICR-MS measurements, which were compared with data obtained from the pure standard (data not shown for proprietary reasons).

Various lipids that highlight different anatomical features were observed in the tissue section, such as sphingomyelin (SM) and phosphatidylcholine (PC) defining lesions in the fibrotic connective tissue (Figure 2A, III). The most abundant of these lipids was ([PC (16:0_16:0) + K]^+^) at *m*/*z* 772.52 (observed exact mass *m*/*z* 772.5250/-0.388 ppm). The liver parenchyma (Figure 2A, IV) was also characterized by PC species, among which [PC (18:0_20:4 + K]^+^) at *m*/*z* 848.55 (observed exact mass *m*/*z* 848.5562/-0.471 ppm) was the most abundant molecular species. The positive ion overlay (Figure 2A, V) clearly shows that compound A is located in the center of the fibrotic lesions, which are considered to be in the bile ducts of the portal areas. This presence could potentially be linked to biliary excretion based on the presence of a series of bile-related species at *m*/*z* 544.24–592.24, which were separated by 16 Da, as previously observed.^16^ These species were identified as TCDCA/TDCA ([M+2Na–H]^+^) at *m*/*z* 544.27, TCA ([M+2Na–H]^+^) at *m*/*z* 560.26, TCDCA/TDCA ([M+2K–H]^+^) at *m*/*z* 576.22, and TCA ([M+2K–H]^+^) at *m*/*z* 592.21. The analysis of sodiated and potassiated bile acid standards was used to confirm the identification (Figure S3A). High mass resolution MS/MS obtained from pure sodiated standards and a compound A-dosed liver tissue section was used to confirm the identification and fragmentation sites (Figure S3B). The potassiated bile acid species were confirmed only by high mass resolution measurements. This identification is further demonstrated by the distribution of the drug and its metabolite colocalizing with bile-related species (Figure S4).

Following analysis in positive ion mode, a consecutive tissue section was analyzed in negative ion mode to determine the identity and distribution of other species present in the tissue section (Figure 2B). The most abundant species detected in negative ion mode were taurine-conjugated bile acids (Figure 2B, section I). Taurocholic acid ([M-H]^−^) at *m*/*z* 514.28 (observed exact mass *m/*z 514.2841/-0.583 ppm) was highly localized in the bile duct lumen and distributed throughout the parenchyma.

In comparison, the bile acids were only localized within the bile duct lumen in healthy rat and dog livers.^16,19^ The fibrotic connective tissue (Figure 2B, II) was once again defined by PC and SM lipids. However, in negative ion mode, they were detected as demethylated ions ([M-CH_3_]^−^). The most abundant of these lipids was ([SM (18:1_16:0) - CH_3_]^−^) at *m*/*z* 687.54 (observed exact mass *m*/*z* 687.5446/ −0.145 ppm). This molecular species has previously been visualized in the necrotic regions of tumors and detected in rat brain tissue extracts.^20,21^ The proposed method for forming these species has previously been discussed.^22,23^ Furthermore, the distribution matches that of its positive ion counterpart ([SM (18:1_16:0) + H]^+^) at *m*/*z* 703.57 (observed exact mass *m*/*z* 703.5748/-0.06 ppm), which correlates with the MTC histological stains. The identification was further confirmed by analyzing a pure standard of SM (18:1_16:0) in both positive and negative ion modes (Figure S5), which match those in previously published material.^23,24^

The parenchyma (Figure 2B, IV) was characterized by ([PI (18:0_20:4) – H]^−^) at *m*/*z* 885.55 (observed exact mass *m*/*z* 885.5501/0.226 ppm). The distribution correlates well with ([PC (18:0_20:4) + K]^+^), which defined this area in positive ion mode. The image also shows that this molecular species uniquely defined the parenchyma and was absent from the areas characterized by the other molecular species. Sulfatides (ST) and hydroxysulfatides (ST–OH) which were previously identified as markers for the bile duct epithelium (Figure 2B, V) were also observed.^16^ The most abundant was [ST–OH (18:1_24:0) - H]^−^) at *m*/*z* 906.64 (observed exact mass *m*/*z* 906.6353/0.772 ppm), which was localized in the middle of the lesions. To further identify the lipids in both polarities, high mass resolution MS/MS measurements were performed (Figures S6–S11). The images obtained from the other biological replicates dosed with compound A are shown in Figure S12.

These features were also highlighted in the H&E stain (Figure 2C, I) and more pronounced in the MTC stain (Figure 2C, II), which shows the cytoplasm of the hepatocytes in red and the fibrotic connective tissue of the lesions indicated by the presence of collagen in blue.^25^ The MTC stain also correlates well with the distribution of the identified SM and PC markers for fibrotic connective tissue (Figure 2A, III and B, II). The insets show an expanded view of the lesions and the areas that were later targeted for high spatial resolution imaging.

**Figure 3:**
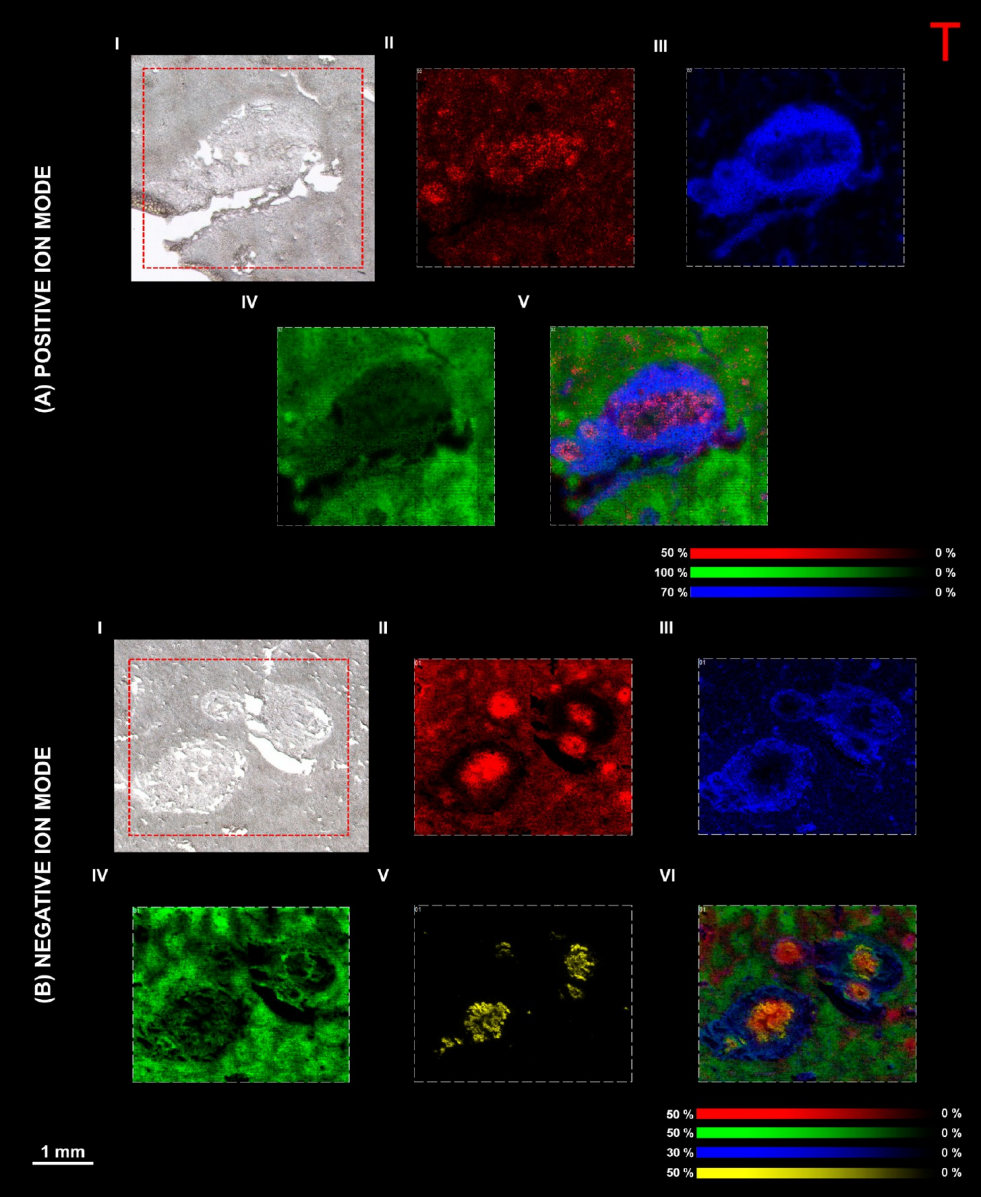
MALDI-MS imaging of compound A–dosed liver tissue (dog 3). (A) MALDI-MS imaging in positive ion mode showing (I) the optical image of the tissue section before matrix application and resulting MALDI-MS images showing the distribution of (II) compound A ([M + H]^+^) at *m*/*z* 502.15, (III) ([PC (32:0) + K]^+^) at *m*/*z* 772.52, (IV) ([PC (38:4) + K]^+^) at *m*/*z* 848.54, and (V) the overlay of selected species. (B) MALDI-MS imaging in negative ion mode showing (I) the optical image of the tissue section before matrix application and resulting MALDI-MS images showing the distribution of (II) taurocholic acid ([M – H]^−^) at *m*/*z* 514.28, (III) ([SM (18:1_16:0) – CH_3_]^−^) at *m*/*z* 687.54, (IV) ([PI (18:0_20:4) – H]^−^) at *m*/*z* 885.55, (V) ([ST–OH (18:1_24:0) – H]^−^) at *m*/*z* 906.64, and (VI) the overlay of selected species (spatial resolution 10 × 10 μm, normalized with TIC).

High spatial resolution MALDI-MS imaging experiments were performed on consecutive tissue sections in both positive and negative ion modes to provide more detail about the lesions. The results from the liver tissue of dogs dosed with compound A are shown in Figure 3.

The high spatial resolution images provide more detail for certain molecular species. For example, images obtained in positive ion mode clearly show that compound A ([M + H]^+^) at *m*/*z* 502.15 (Figure 3A, II) was elevated in the center of the lesion. The negative ion image of [ST–OH (18:1_24:0) - H]^−^ at *m*/*z* 906.64 (Figure 3B, V) shows that the bile duct epithelium was thickened and irregular, consistent with observations in Figures 1A, III and IV. In contrast, the bile duct in healthy dog liver appeared as a thin band around the bile duct lumen.^16^ The high spatial resolution images also provide more detailed views of the bile duct lumen, the fibrotic connective tissue, and the parenchyma in both polarities.

### Multimodal Imaging of Compound B Dosed Liver Tissue Sections

**Figure 4:**
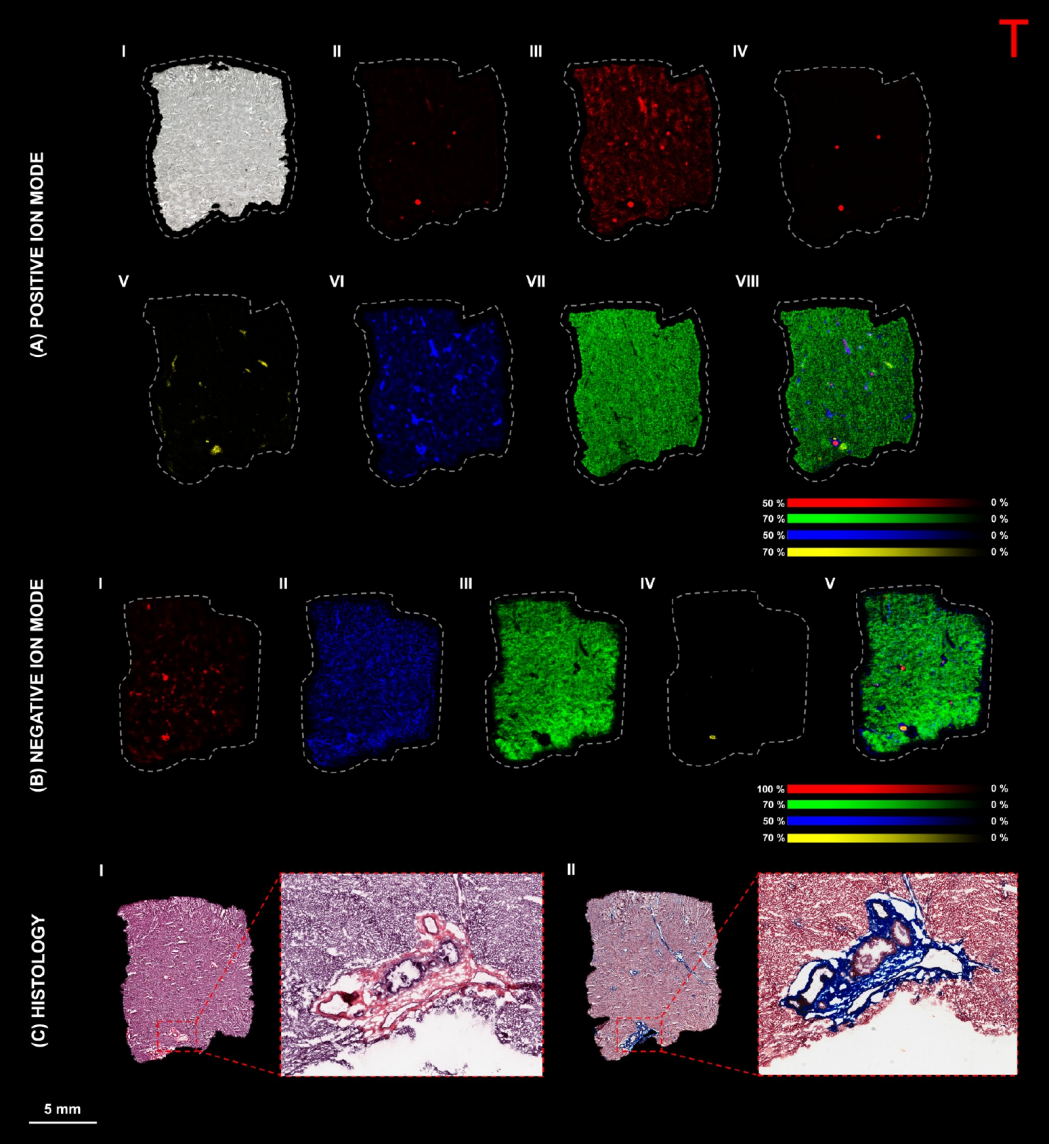
MALDI-MS imaging of compound B-dosed liver tissue (dog 1). (A) MALDI-MS imaging in positive ion mode showing (I) an optical image of the tissue section before matrix application and resulting MALDI-MS images showing the distribution of (II) desmethyl metabolite ([M + H]^+^) at *m*/*z* 502.15, (III) compound B ([M + H]^+^) at *m*/*z* 516.16, (IV) *N*-oxide metabolite ([M + H]^+^) at *m*/*z* 532.16, (V) heme ([M]^+^) at *m*/*z* 616.17, (VI) ([PC (32:0) + K]^+^) at *m*/*z* 772.52, (VII) ([PC (38:4) + K]^+^) at *m*/*z* 848.54, and (VIII) the overlay of selected species. (B) MALDI-MS imaging in negative ion mode showing the distribution of (I) taurocholic acid ([M – H]^−^) at *m*/*z* 514.28, (II) ([SM (18:1_16:0) – CH_3_]^−^) at *m*/*z* 687.54, (III) ([PI (18:0_20:4) – H]^−^) at *m*/*z* 885.55, (IV) ([ST–OH (18:1_24:0) – H]^−^) at *m*/*z* 906.64, and (V) the overlay of selected species (spatial resolution 30 × 30 μm, normalized with TIC). (C) Histology of consecutive sections stained with (I) H&E and (II) MTC protocols (insets show areas chosen for high spatial resolution imaging).

Following the analysis of livers from dogs dosed with compound A, the livers of those dosed with compound B were analyzed in the same way (Figure 4).

The optical images of the tissue section (Figure 4A, I) indicate that the lesions were smaller in the liver dosed with compound B, with diameters ranging from 0.2 to 0.5 mm, compared to those in the liver treated with compound A, which measured between 0.5 to 2 mm. The liver dosed with compound B showed the presence of compound B at *m*/*z* 516.16 (Figure 4A, III) and its two metabolites, an N-desmethyl metabolite at *m*/*z* 502.15 (Figure 4A, II) and most likely an *N*-oxide metabolite at *m*/*z* 532.16 (Figure 4A, IV). Compound B was distributed throughout the liver tissue section but more concentrated in the center of the lesions, whereas the *N*-oxide and N-desmethyl metabolites were primarily localized in the center of the lesions. The identities of compound B and the N-desmethyl metabolite (compound A) were confirmed by MALDI-MS/MS and MALDI-FTICR-MS, which matched the data obtained from the pure standards. However, the identity of the *N*-oxide metabolite was established solely through high mass accuracy measurements. This *N*-oxide metabolite and the phenolic metabolite mentioned above were previously identified during metabolite identification studies (data not shown).

As with the previous images, the endogenous species were also found to be localized to specific areas of the liver tissue. Monitoring a combination of the distribution of bile acids and heme ([M]^+^) at *m*/*z* 616.18 (observed exact mass 616.1766 /-0.325 ppm), the smaller bile ducts and minor portal triads could be identified (Figure 4A, V). The image clearly shows blood vessels of varying sizes throughout the tissue. The fibrotic connective tissue was again highlighted by ([PC (16:0_16:0) + K]^+^) at *m*/*z* 772.52 (Figure 4A, VI), which distinctly defines the lesions. As with compound A, the surrounding parenchyma was characterized by (PC (18:0_20:4 + K]^+^) at *m*/*z* 848.55 (Figure 4A, VII). The positive ion overlay (Figure 4A, VIII) clearly demonstrates how all the selected species colocalize, clearly defining the different anatomical regions. The image shows the drug and metabolites residing in the center of the lesions, which are considered to be bile ducts in portal areas, surrounded by fibrotic connective tissue. The overlay also shows that lesions were often located near blood vessels as most of the blood vessels are part of the hepatic triad (hepatic artery, hepatic portal vein, and bile ducts).

Negative ion mode analysis was performed on a consecutive tissue section to provide complementary information about the other endogenous species present in the liver of dosed dogs. Similar to the compound A–dosed tissue, the most abundant species detected in negative ion mode from compound B–dosed tissue were bile acids, specifically taurine-conjugated bile acids, such as taurochenodeoxycholic/taurodeoxycholic acid (TCDCA/TDCA) and taurocholic acid (TCA), which is expected, as dogs produce predominantly taurine-conjugated bile acids.^26^ The distribution of taurocholic acid at *m*/*z* 514.28 (Figure 4B, I) was localized in the center of the lesions but also dispersed throughout the tissue. Species related to the fibrotic connective tissue, such as ([SM (18:1_16:0) – CH_3_]^−^) at *m*/*z* 687.54 (Figure 4B, II), and those related to the parenchyma ([PI (18:0_20:4) – H]^−^) at *m*/*z* 885.55 (Figure 4B, III), were also found in negative ion mode. The images were similar to those in positive ion mode and those observed in the compound A-dosed tissue. The inner part of the lesion was highlighted by ([ST–OH (18:1_24:0) – H]^−^) at *m*/*z* 906.64 (Figure 4B, IV), again indicating the bile ducts as the center of these lesions. The overlay (Figure 4B, V) shows the colocalization of the selected species, which was the target for high spatial resolution imaging. Consecutive tissue sections were histologically stained for reference (Figure 4C, I and II). Images of the selected molecular species obtained from the other biological replicate dosed with compound B are shown in Figure S13.

**Figure 5:**
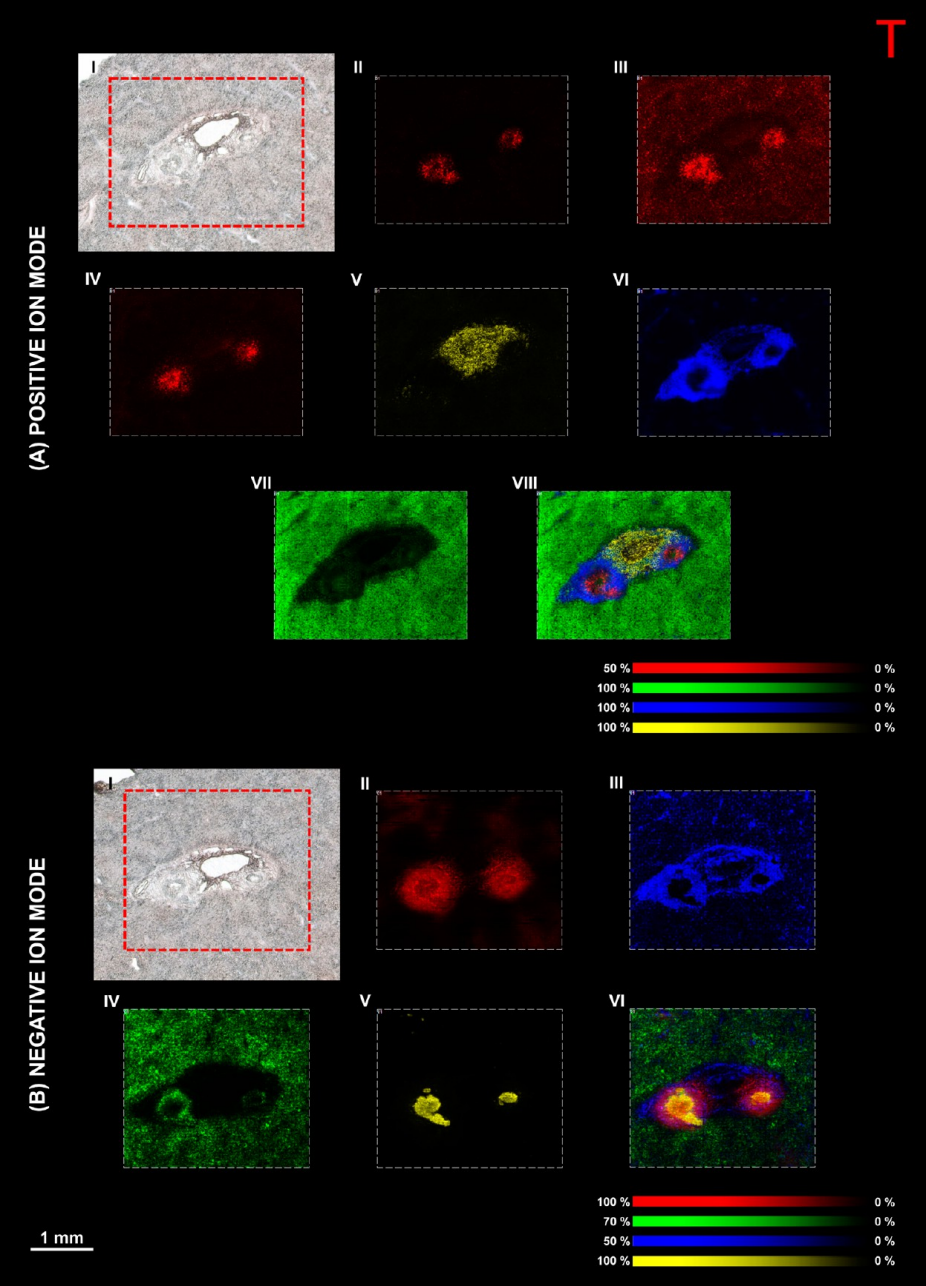
MALDI-MS imaging of compound B–dosed liver tissue (dog 2). (A) MALDI-MS imaging in positive ion mode showing (I) the optical image of tissue section before matrix application and resulting MALDI-MS images showing the distribution of (II) desmethyl metabolite ([M + H]^+^) at *m*/*z* 502.15, (III) compound B ([M + H]^+^) at *m*/*z* 516.16, (IV) *N*-oxide metabolite ([M + H]^+^) at *m*/*z* 532.16, (V) heme ([M]^+^) at *m*/*z* 616.17, (VI) ([PC (32:0) + K]^+^) at *m*/*z* 772.52, (VII) ([PC (38:4) + K]^+^) at *m*/*z* 848.54, and (VIII) the overlay of selected species. (B) MALDI-MS imaging in negative ion mode showing (I) the optical image of tissue section before matrix application and resulting MALDI-MS images showing the distribution of (II) taurocholic acid ([M – H]^−^) at *m*/*z* 514.28, (III) ([SM (18:1_16:0) – CH_3_]^−^) at *m*/*z* 687.54, (IV) ([PI (18:0_20:4) – H]^−^) at *m*/*z* 885.55, (V) ([ST–OH (18:1_24:0) – H]^−^) at *m*/*z* 906.64, and (VI) the overlay of selected species (spatial resolution 10 × 10 μm, normalized with TIC).

High spatial resolution MALDI-MS imaging experiments were subsequently performed on a consecutive liver tissue section from the second dog dosed with compound B (Figure 5).

The high spatial resolution images confirm earlier findings and provide more detailed information about the spatial distribution of the drug and its metabolites. They clearly show that compound B, the N-desmethyl metabolite, and *N*-oxide metabolites were colocalized in the center of the small lesions (Figure 5A, II–IV respectively). The blood vessel located between the two small lesions was highlighted by the distribution of heme at *m*/*z* 616.17 (Figure 5A, V). The positive and negative ion images provide a more detailed view of the fibrotic part of the lesions (Figure 5A, VI and B, III respectively), surrounding compound B, its metabolites, and the bile acids. Lesions were located in portal areas adjacent to normal liver parenchyma (Figure 5A, VII and B, IV respectively). The negative ion mode images of a smaller bile duct show the sulfatide (Figure 5B, V) appearing to block/constrict the center of the bile duct/lesion, as seen from the local hyperplasia of the epithelium, which explains the high concentration of compound B, its metabolites, and the bile acids in the center of the lesions. The overlay of the selected species (Figure 5B, VI) shows their complementarity. A healthy dog liver tissue section was used as a negative control and compared with the two dosed tissue samples (Figure S14A-C). The MALDI-MS images clearly show the absence of the two candidate compounds and their metabolites in the negative control tissue (Figure S14, II–IV), a finding also reflected in the corresponding intensity box plots. Additionally, the MALDI-MS images show that the connective tissue molecular marker (Figure S14, V) and parenchyma molecular marker (Figure S14, VI) were more prevalent in the compound A-dosed liver compared to both control tissue and tissue dosed with compound B. This increase was also reflected in the ROC curves (Figure S15A, B), which show the connective tissue marker to be more abundant in the compound A–dosed tissue in comparison with the control tissue (AUC = 0.850) and the tissue treated with compound B (AUC = 0.799). Similar observations were made with the parenchyma marker (AUC = 0.865 and 0.896, respectively).

### Comparison of Liver Pathology

**Figure 6:**
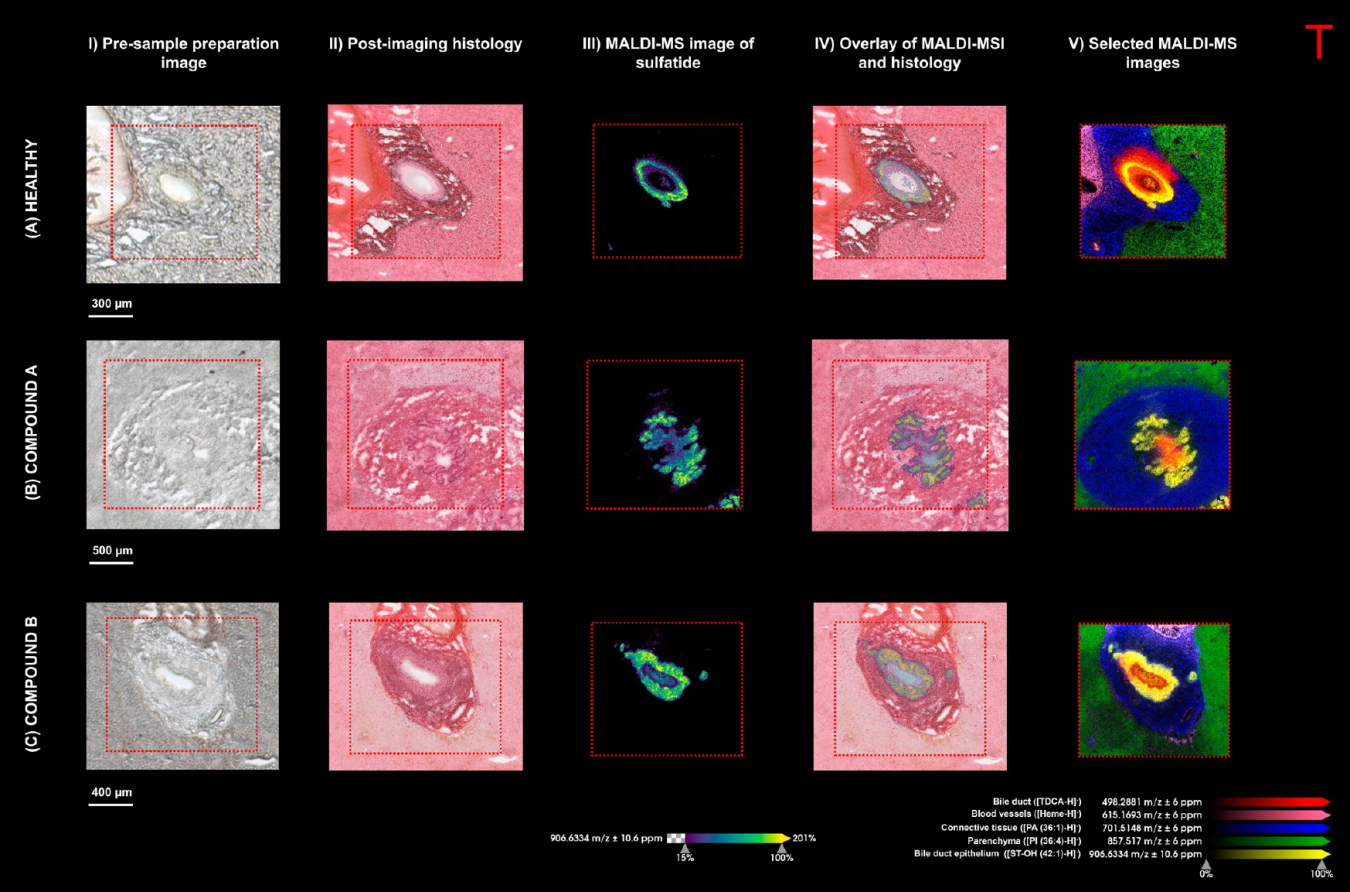
Comparison of the liver pathology in (A) healthy dog liver tissue, (B) compound A-dosed dog liver tissue, and (C) compound B-dosed dog liver tissue. (I) Optical image of tissue section prior to matrix application, (II) postimaging histology of tissue sections, (III) MALDI-MS images showing the distribution of the bile duct epithelium marker ([ST–OH (18:1_24:0) – H]^−^) at *m*/*z* 906.6334, and (IV) overlay of the MALDI-MSI data and the postimaging stain. (V) Overlay of selected molecular species that highlight the different anatomical features of the portal area (spatial resolution of 5 × 5 μm and normalized with the TIC).

To get a better understanding of the effects of the candidate drug compounds on the liver, a comparison with healthy control tissue from a previous study was performed (Figure 6).

The optical images (Figure 6A-C, I) and the postimaging histology (Figure 6A-C, II) show the areas selected for high spatial resolution imaging in negative ion mode. The distribution of the molecular marker for the bile duct epithelium ([ST–OH (18:1_24:0) - H]^−^) at *m*/*z* 906.6334), was monitored to observe the changes resulting from the administered drug compounds. The MALDI-MS images of the healthy control liver tissue sections (Figure 6A, III) show a thin band (duct) distribution. In contrast, the dosed liver tissue sections show drug-induced changes to the bile duct epithelium: The compound A-dosed liver tissue section (Figure 6B, III) shows an irregular and hyperplastic bile duct, whereas the compound B-dosed liver tissue section (Figure 6C, III) of a larger bile duct shows a thickened ring-like (duct) structure. Overlaying the distribution of this molecular marker onto the postimaging histological image (Figure 6A-C, IV) shows its colocalization with the bile duct region.

Another observed effect of the candidate drug compounds was an increase in peri-ductal connective tissue (fibrosis). The MALDI-MS images show the average connective tissue thickness in the healthy control tissue was approximately 130 μm. In comparison, the connective tissue of compound A-dosed tissue showed increased collagen (fibrosis) and an average thickness of 470 μm. The connective tissue of compound B-dosed tissue showed less fibrosis, with an average thickness of around 170 μm. The changes observed in the tissue were further exemplified when viewing the distribution of the identified molecular markers for the bile duct/epithelium, blood vessels, connective tissue, and parenchyma (Figure 6A-C, V).

## Conclusions

A multimodal approach using a combination of MALDI-MSI and histological staining was used to investigate pathological changes, specifically lesions resulting from a candidate drug (compound B) and its metabolite (compound A). MALDI-MSI allowed for the simultaneous monitoring of the distribution of the drug, its metabolites, and the endogenous lipid species associated with anatomical features. The images revealed that the drugs and their metabolites were distributed throughout the liver but primarily located in the center of the lesions, while the lipids highlighted drug-induced metabolic changes in the connective tissue and bile ducts.

High spatial resolution MALDI-MS imaging, paired with histological stains, provided a clearer view of these changes. The most substantial changes were observed in the livers of dogs dosed with compound A, which showed increased connective tissue fibrosis and bile duct hyperplasia. In contrast, dogs dosed with compound B primarily showed bile duct degeneration and regeneration, as evidenced by a thickened bile duct epithelium. This study highlights the potential benefit of spatial biology technology for use in toxicological studies. The MALDI-MSI results complemented the histological staining and may offer insights into the underlying mechanisms of action.

## Supporting Information Available

MALDI-MS spectra of compound A and compound B standards, on-tissue MALDI matrix evaluation tests, high-resolution MALDI-MS and MS/MS identification of positive ion bile acids, MALDI-MS images showing potential evidence of biliary excretion, identification of positive and negative lipids using high mass resolution MALDI-MS/MS, MALDI-MSI of biological replicates dosed with compound A and compound B, comparison of negative control liver tissue and dosed liver tissue with intensity box plots and corresponding ROC curves of connective tissue and parenchyma molecular markers (PDF)
