# Supplementary material for: Investigation into Drug-Induced Liver Damage Using Multimodal Mass Spectrometry Imaging
Source: J Am Soc Mass Spectrom. 2025 Jan 17;36(2):265–76. doi: 10.1021/jasms.4c00313 (PMC11808762; doi:10.1021/jasms.4c00313)
Supplement: Supplementary file 1 — js4c00313_si_001.pdf [file js4c00313_si_001.pdf]

Supporting Information

Investigation into drug-induced liver damage using multi-modal mass spectrometry imaging

Bryn Flinders[1], Lennart Huizing[1†], Bhanu Singh[2‡], Heng-Keang Lim[2⸸], Marjolein van Heerden[3], Filip Cuyckens[3], Ron M. A. Heeren[1], Rob J. Vreeken[1,3*]

[1.] Maastricht MultiModal Molecular Imaging Institute (M4i), Division of Imaging Mass Spectrometry, Maastricht University, Universiteitssingel 50, 6229 ER Maastricht, The Netherlands

[2.] Janssen Research & Development, 1400 McKean Rd, Spring House, PA, 19477, USA

[3.] Janssen Research & Development, Turnhoutseweg 30, 2340 Beerse, Belgium

[*]Address reprint requests to Dr. Rob J. Vreeken, Maastricht MultiModal Molecular Imaging Institute (M4i), Division of Imaging Mass Spectrometry, Maastricht University, Universiteitssingel 50, 6229 ER Maastricht, The Netherlands, telephone +31883887835, e-mail r.vreeken@maastrichtuniversity.nl

Table of Contents

Figure S1. MALDI-MS profiling of compound A and B 2
Figure S2. On-tissue matrix optimization tests 3
Figure S3. Identification of positive bile species 4
Figure S4. Evidence of possible biliary excretion 5
Figure S5. MALDI-MS/MS of SM (18:1_16:0) standard in positive and negative ion mode 6
Figure S6-11. Comparison of MALDI-MS/MS from pure standards and tissue 7-12
Figure S12. MALDI-MS imaging of compound A dosed biological replicates 13
Figure S13. MALDI-MS imaging of compound B dosed biological replicates 14
Figure S14. Comparison of dosed tissue with control tissue 15
Figure S15. Comparison of ROC curves for lipid markers 16

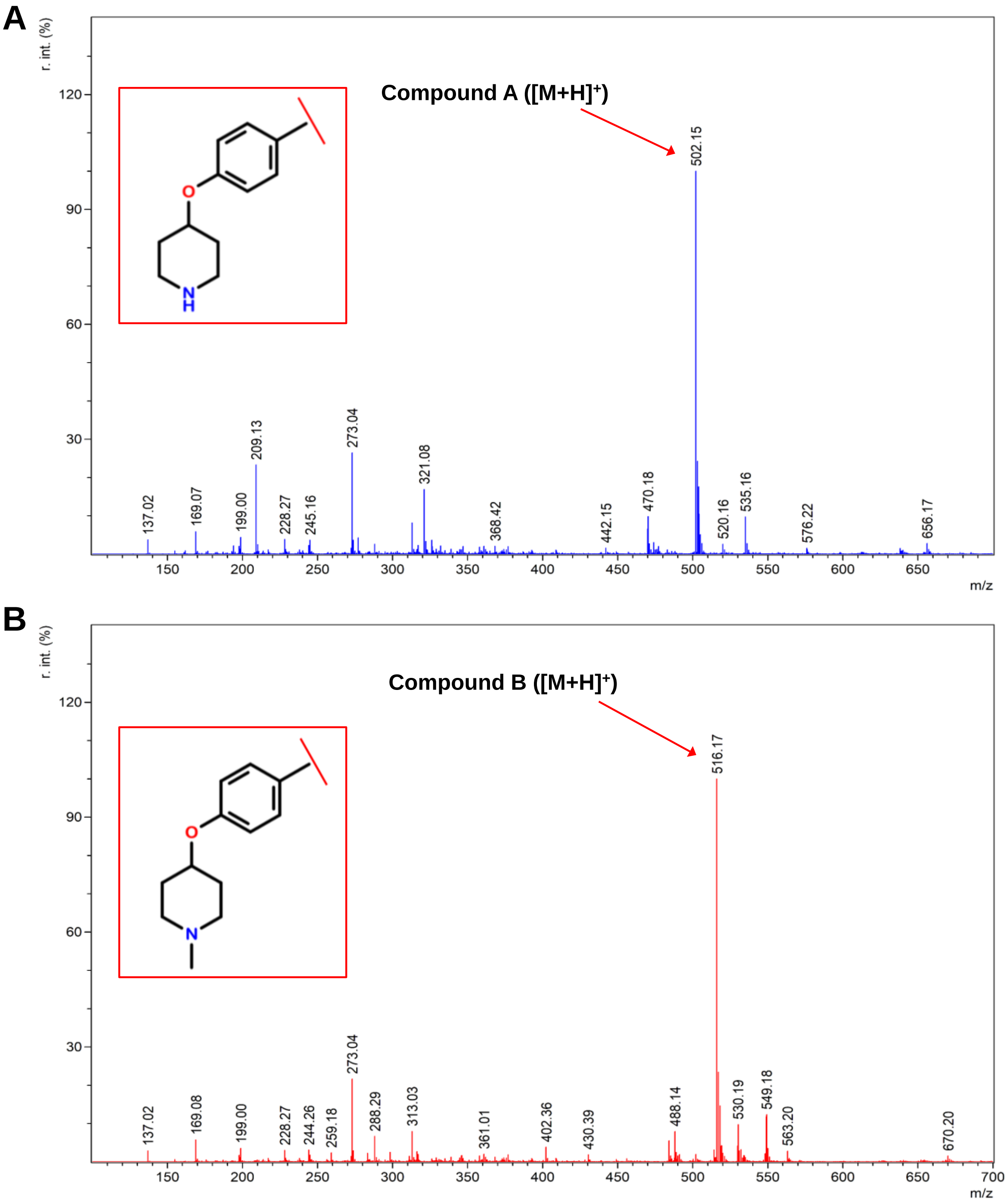

Figure S1. MALDI-MS of compounds A and B. MALDI-MS spectra of A) compound A showing the protonated species at *m/z* 502.15 and B) protonated species of compound B at *m/z* 516.17 (inserts show the partial structures of the two compounds). The standards (100 µg/mL in 70% MeOH) were mixed in a 1:1 v/v ratio with the MALDI matrix (15 mg/mL DHB in 70% MeOH with 0.2% TFA) and spotted onto a target plate for analysis.

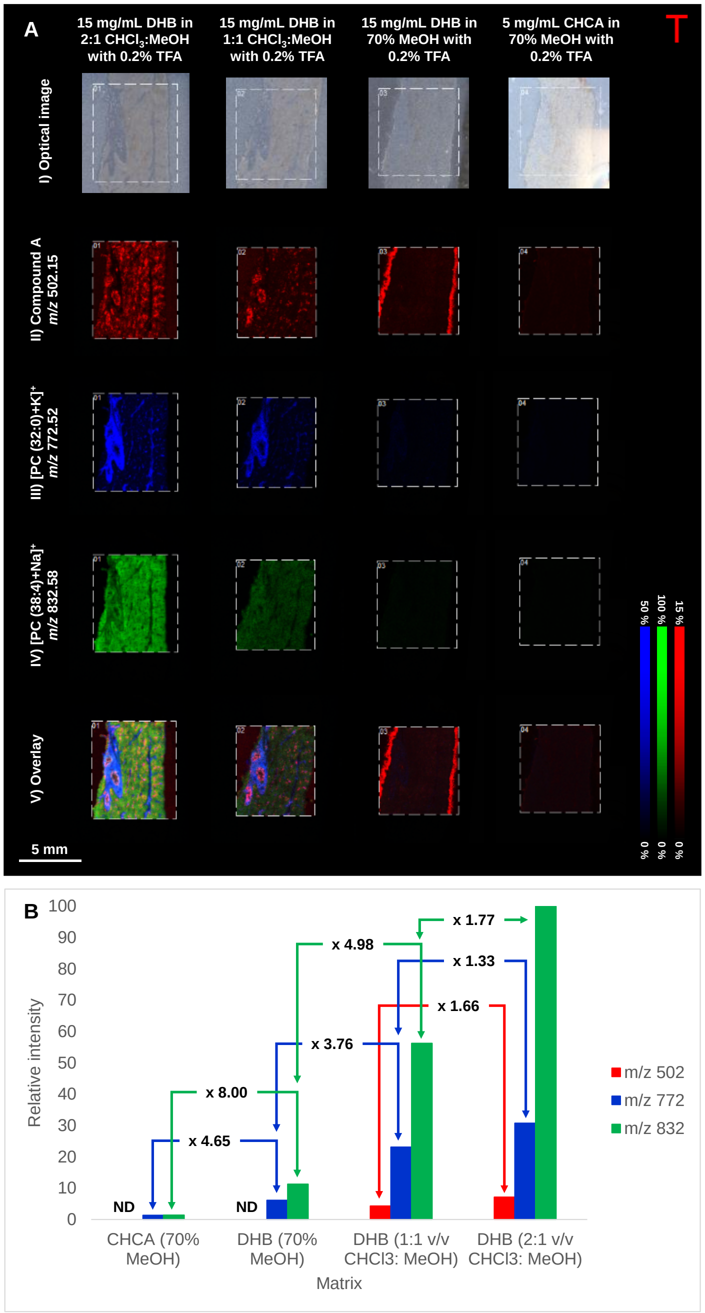

Figure S2. On-tissue matrix evaluation tests. A) Compound A-dosed tissue coated with 5 mg/mL CHCA in 70% MeOH with 0.2% TFA, 15 mg/mL DHB in 70% MeOH with 0.2% TFA, 15 mg/mL DHB in 1:1 $CHCl_3$: MeOH (v/v) with 0.2% TFA, and 15 mg/mL DHB in 2:1 $CHCl_3$: MeOH (v/v) with 0.2% TFA. I) Optical images of the samples following the application of the above matrices. The MALDI-MS images show the distribution of II) compound A ($[M+H]^+$) at *m/z* 502.15, III) connective tissue marker $[PC (32:0)+K]^+$ at *m/z* 772.52, IV) parenchyma marker $[PC (38:4)+Na]^+$ at *m/z* 832.58, and V) overlay of the selected masses (spatial resolution 50 × 50 µm, normalized with TIC). B) Average intensity of the selected masses obtained from regions of interest within the tissue boundaries.

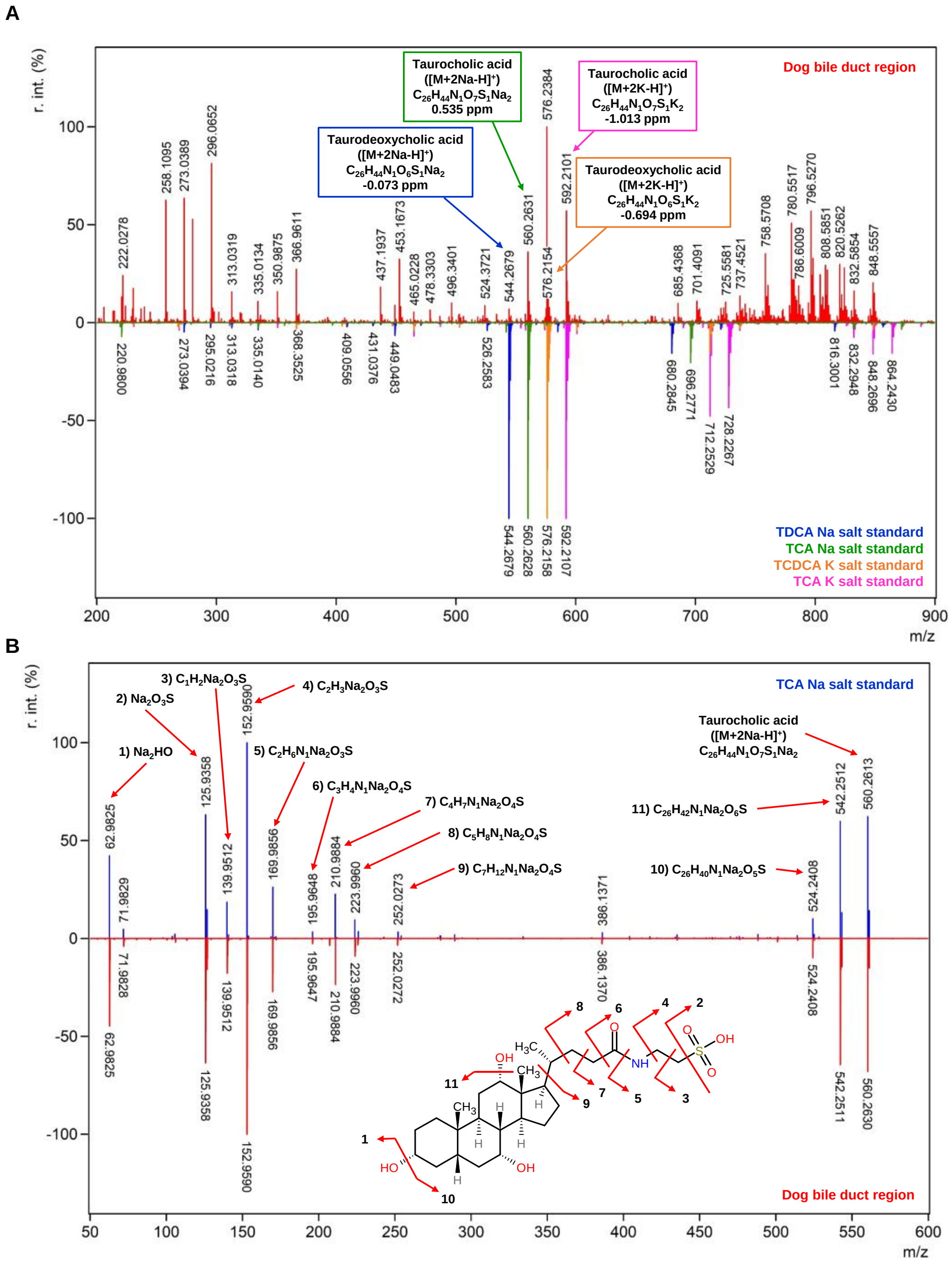

Figure S3. Identification of positive ion bile acid species. A) MALDI-MS spectra obtained from the center of a lesion of a dog liver dosed with compound A compared with sodiated and potassiated taurine conjugated bile acid standards. B) MALDI-MS/MS spectra of a sodiated taurocholic acid standard compared with that obtained from the center of a lesion of a dog liver dosed with compound A (the insert shows the structure of taurocholic acid and the potential fragmentation sites determined high mass resolution MSMS).

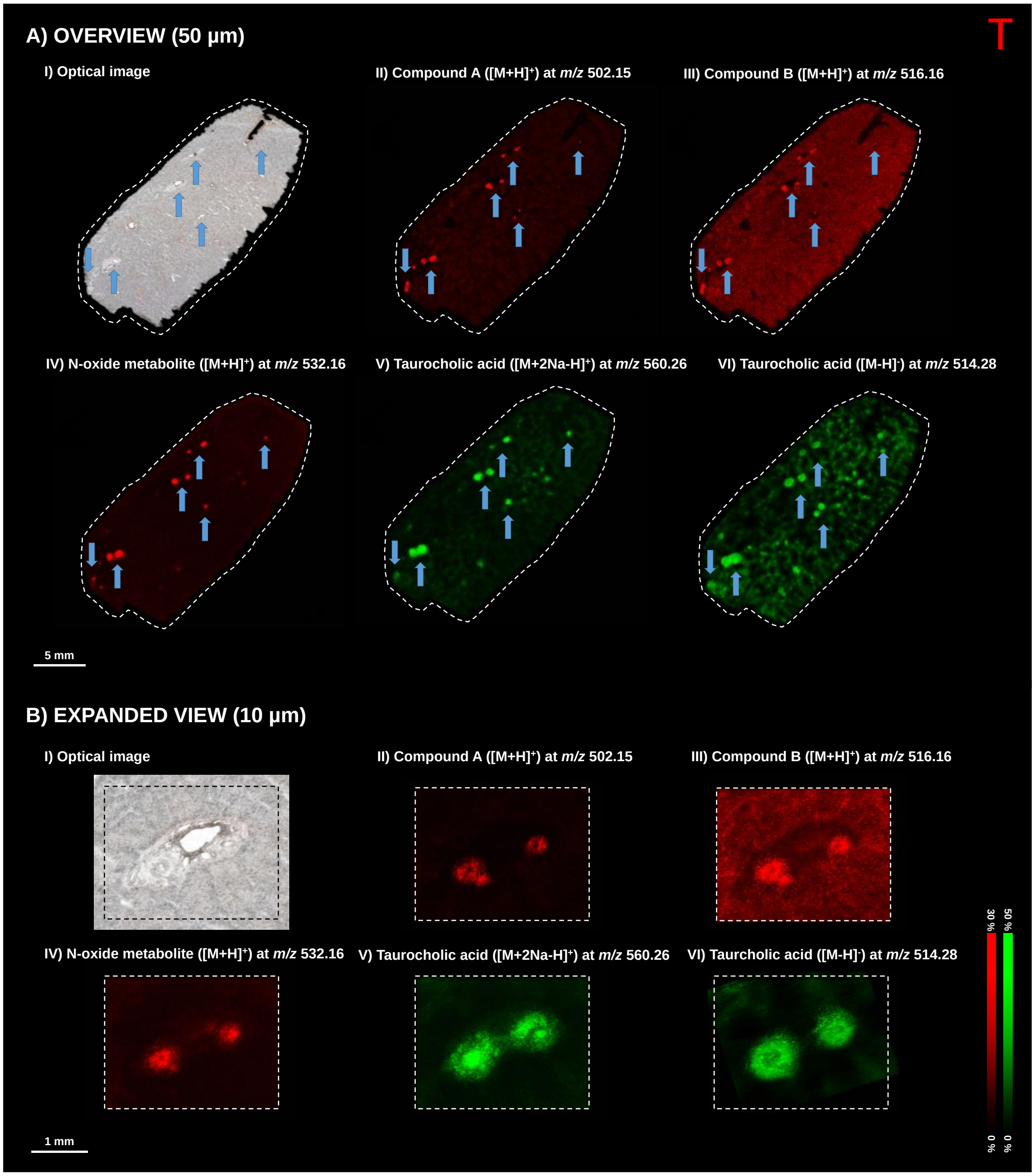

Figure S4. Evidence of possible biliary excretion of drug and metabolites. A) Overview and B) an expanded view of compound B-dosed tissue. I) Optical image of tissue section before matrix application and MALDI-MS images showing the distribution of II) compound B desmethyl metabolite ($[M+H]^+$) at *m/z* 502.15, III) compound B ($[M+H]^+$) at *m/z* 516.16, IV) compound B N-oxide metabolite ($[M+H]^+$) at *m/z* 532.16, V) taurocholic acid ($[M+2Na-H]^+$) at *m/z* 560.26 in positive ion mode and VI) taurocholic acid ($[M-H]^-$) at *m/z* 514.28 in negative ion mode from a consecutive tissue section (spatial resolution as stated, normalized with TIC).

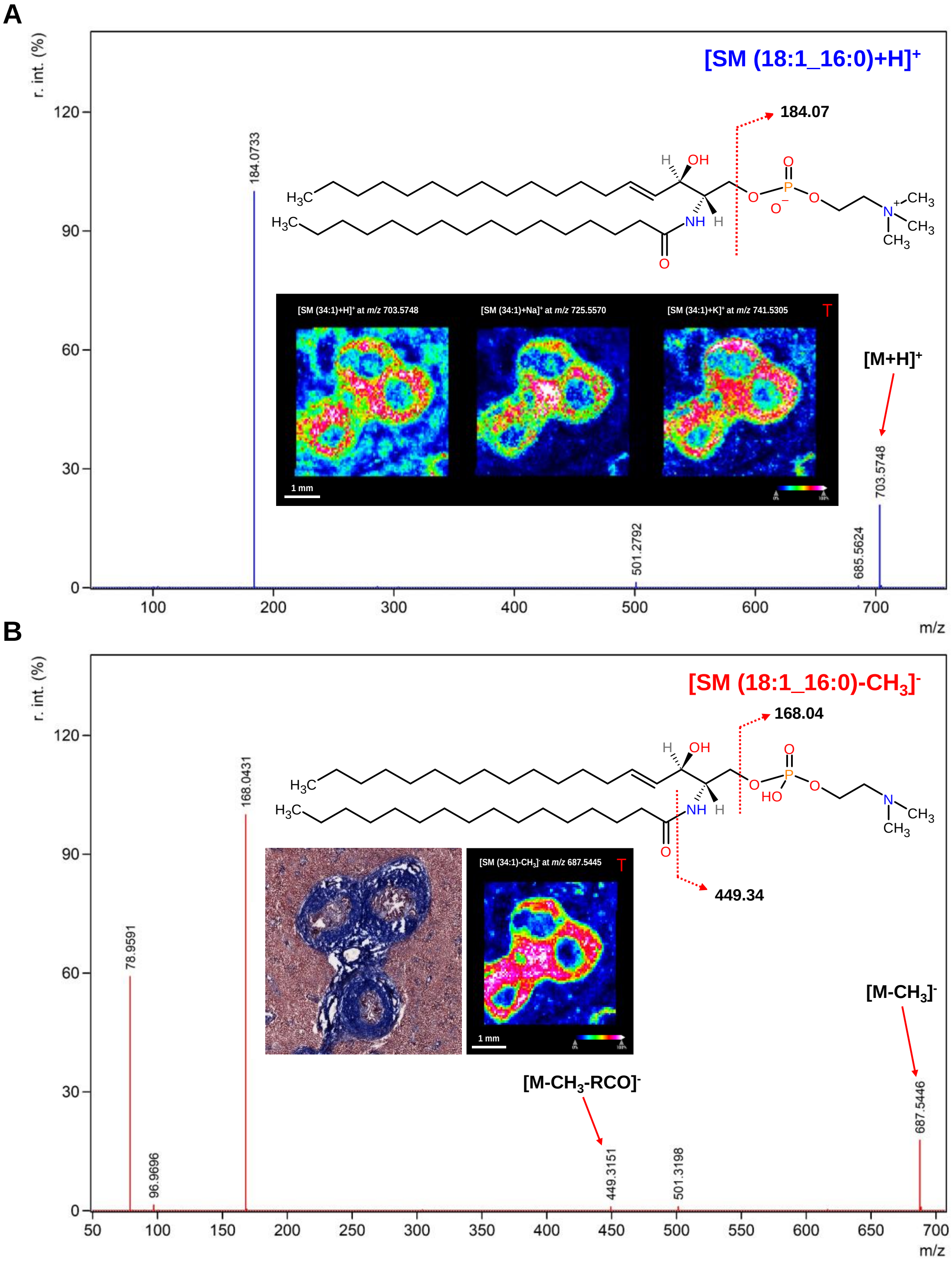

Figure S5. MALDI-MS/MS spectra of SM (18:1_16:0) lipid standard in A) positive ion mode and B) negative ion mode. Inserts show the distribution of these species in the liver tissue sections, the Masson's trichrome stain of a consecutive tissue section, and annotated structures showing the associated fragments.

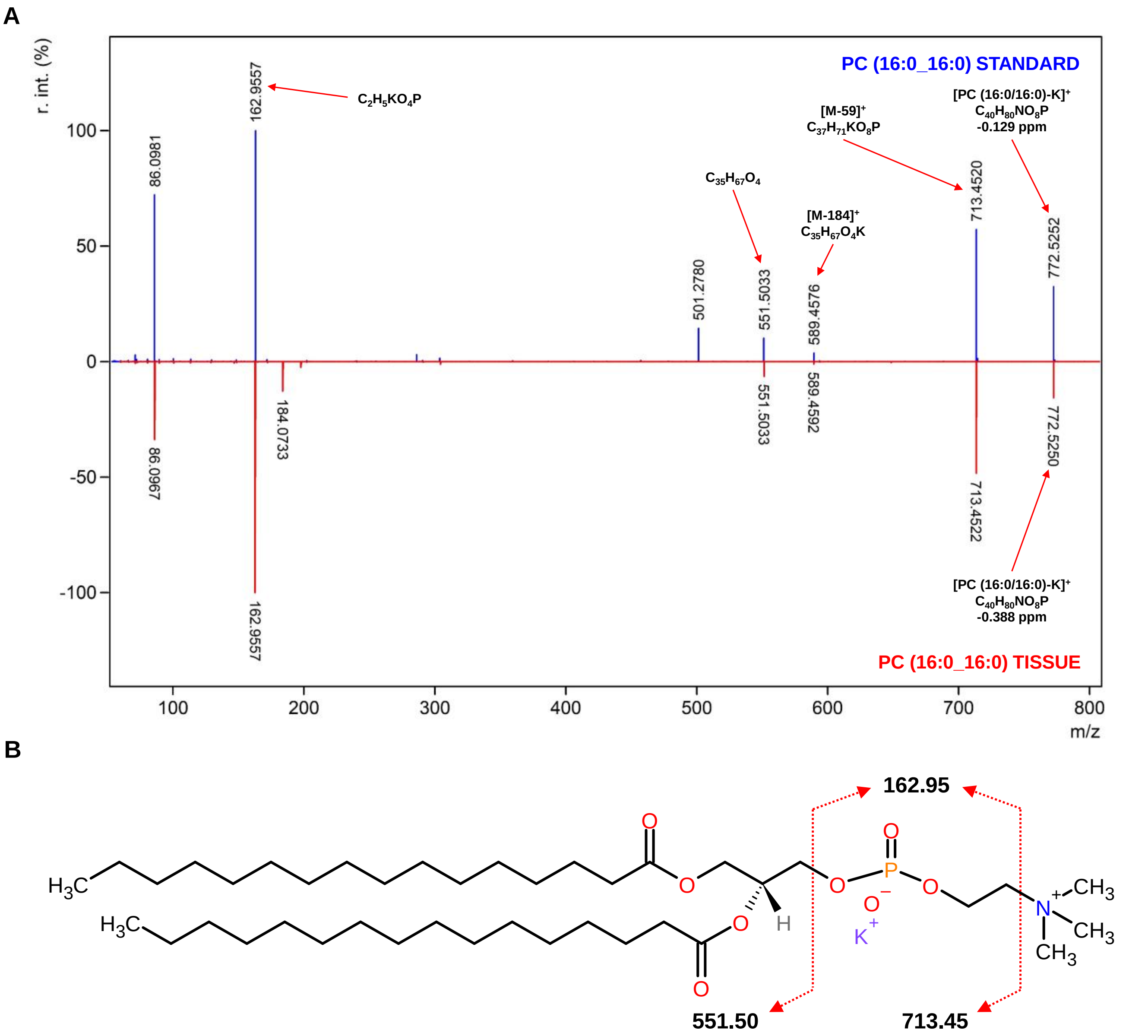

Figure S6. MALDI-MS/MS of PC 16:0_16:0 in positive ion mode. A) MALDI-MS/MS of the pure standard compared to that obtained from the liver tissue sections. B) Structure of PC 16:0_16:0 annotated with the locations of fragmentation.

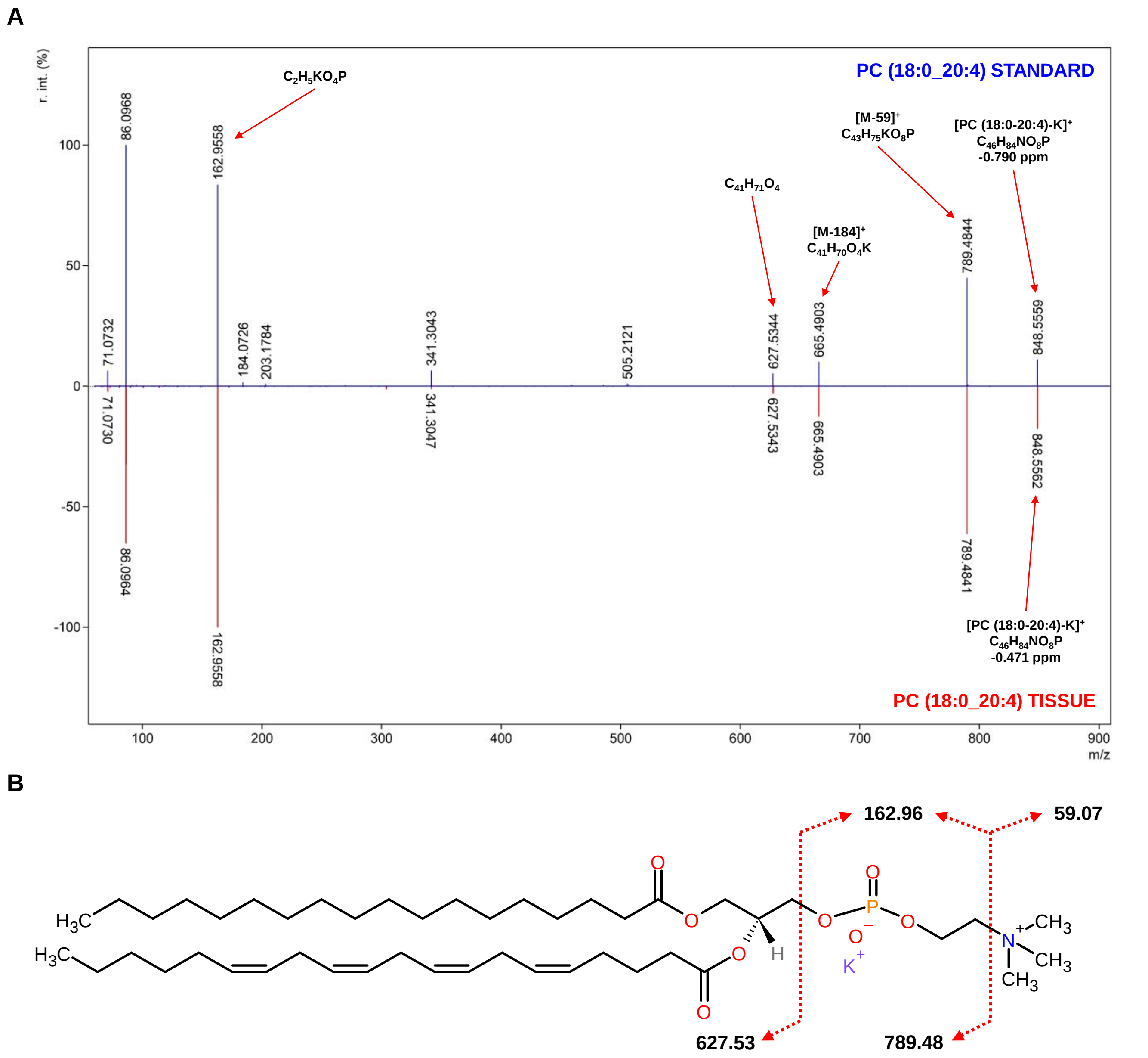

Figure S7. MALDI-MS/MS of PC 18:0_20:4 in positive ion mode. A) MALDI-MS/MS of the pure standard compared to that obtained from the liver tissue sections. B) Structure of PC 18:0_20:4 annotated with the locations of fragmentation.

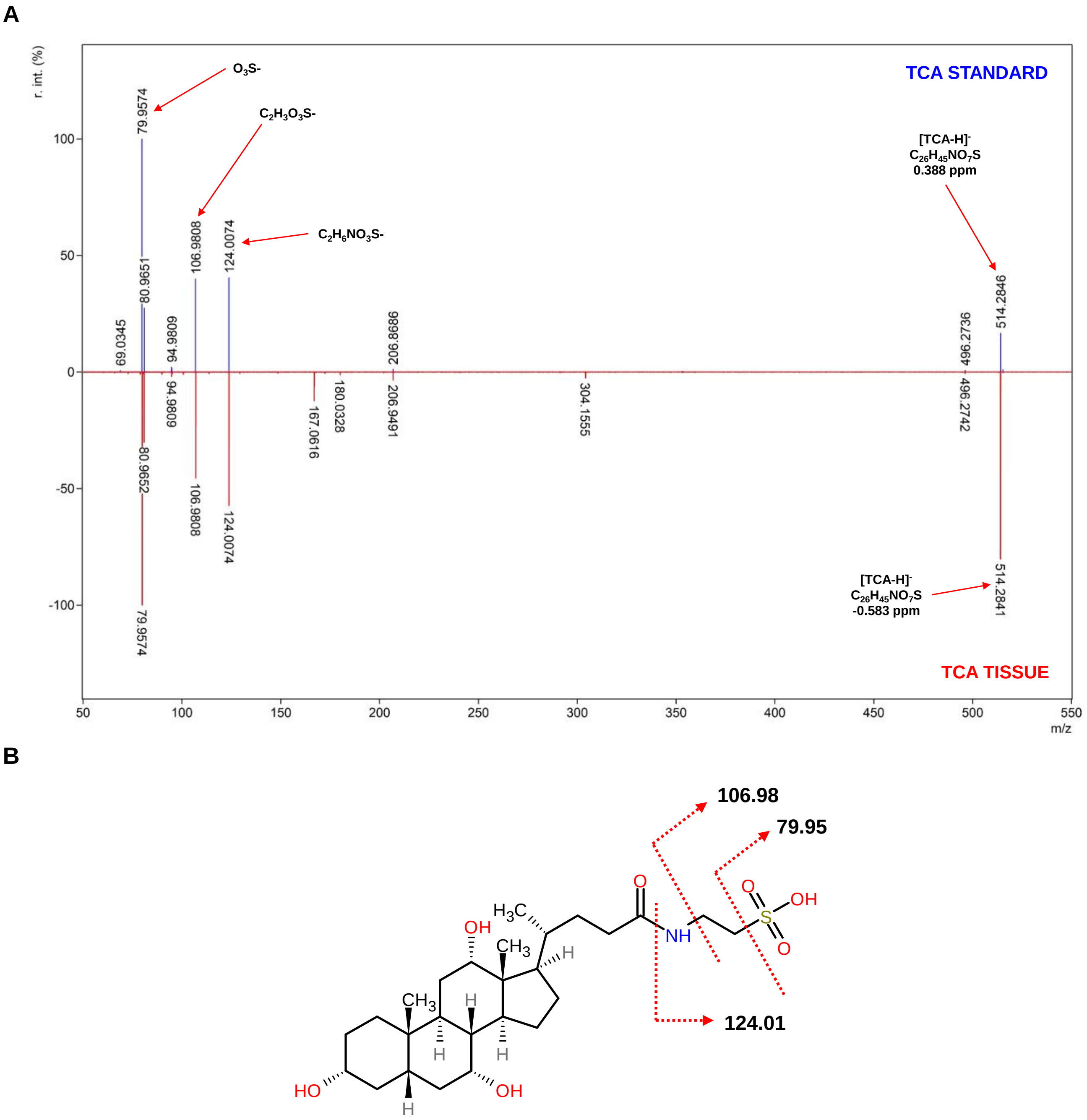

Figure S8. MALDI-MS/MS of TCA in negative ion mode. A) MALDI-MS/MS of the pure standard compared to that obtained from the liver tissue sections. B) Structure of TCA annotated with the locations of fragmentation.

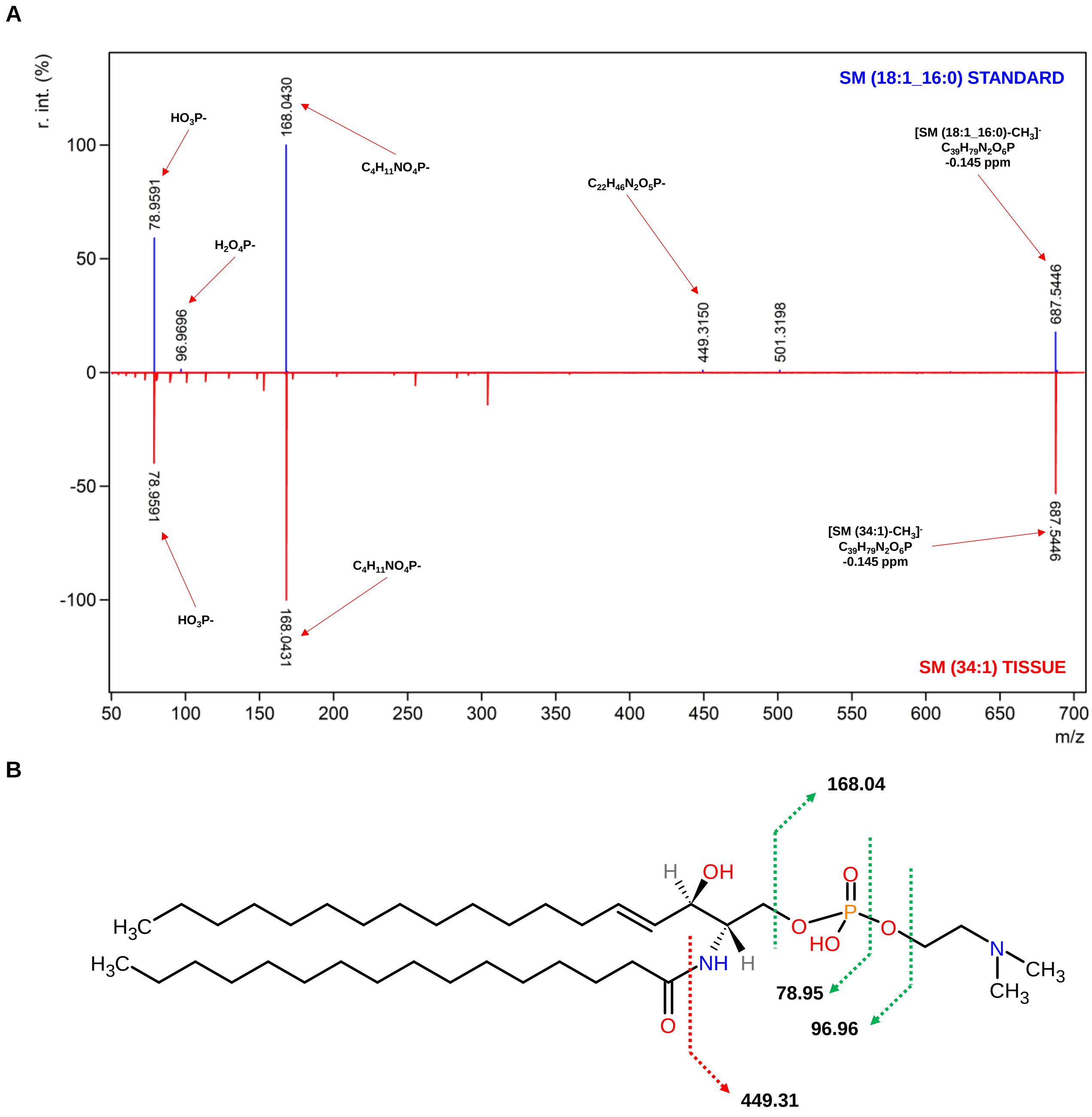

Figure S9. MALDI-MS/MS of SM 16:0_18:1 in negative ion mode. A) MALDI-MS/MS of the pure standard compared to that obtained from the liver tissue sections. B) Structure of SM 18:1_16:0 annotated with the locations of fragmentation.

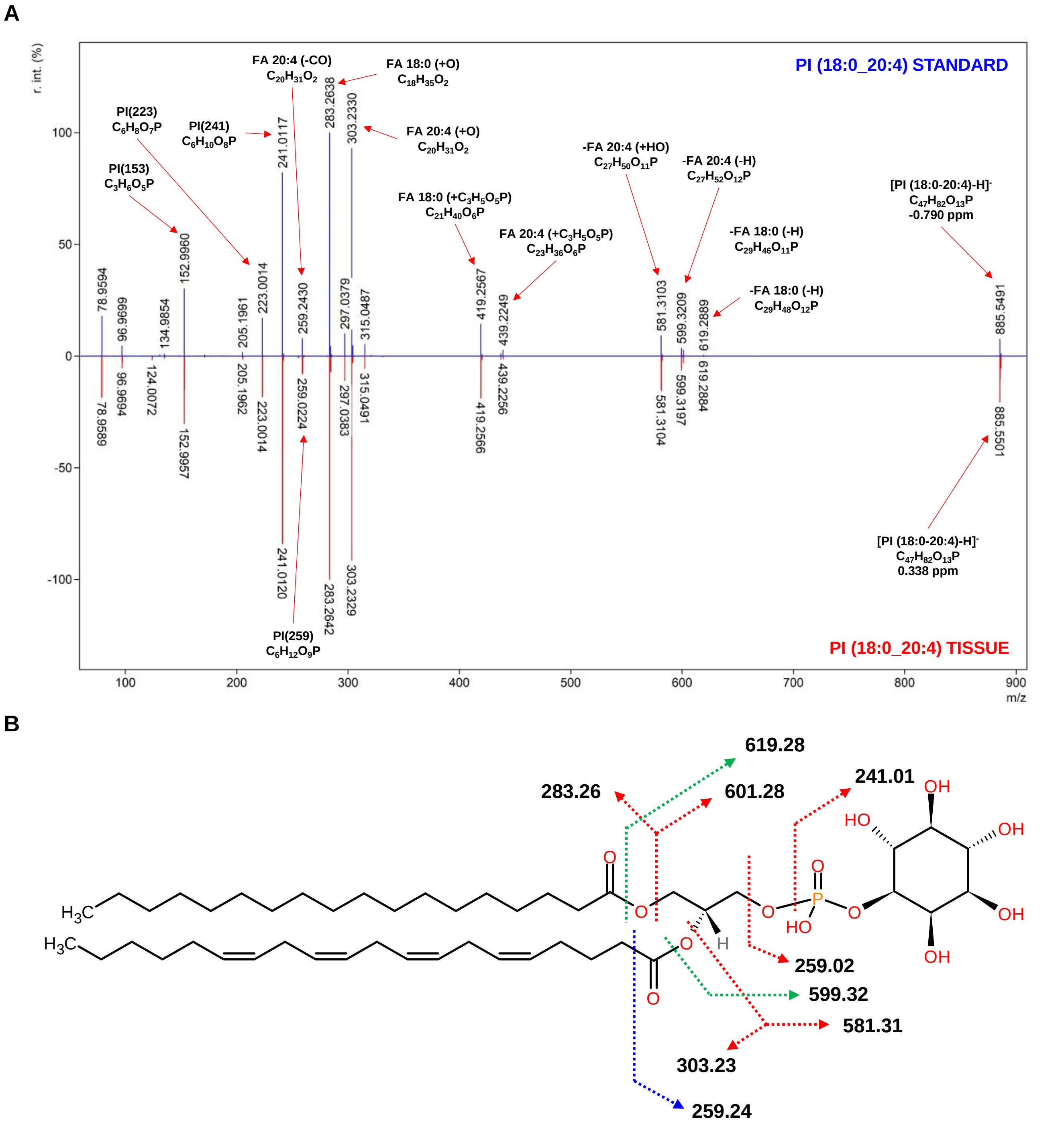

Figure S10. MALDI-MS/MS of PI (18:0_20:4) in negative ion mode. A) MALDI-MS/MS of the pure standard compared to that obtained from the liver tissue sections. B) Structure of PI (18:0_20:4) annotated with the locations of fragmentation.

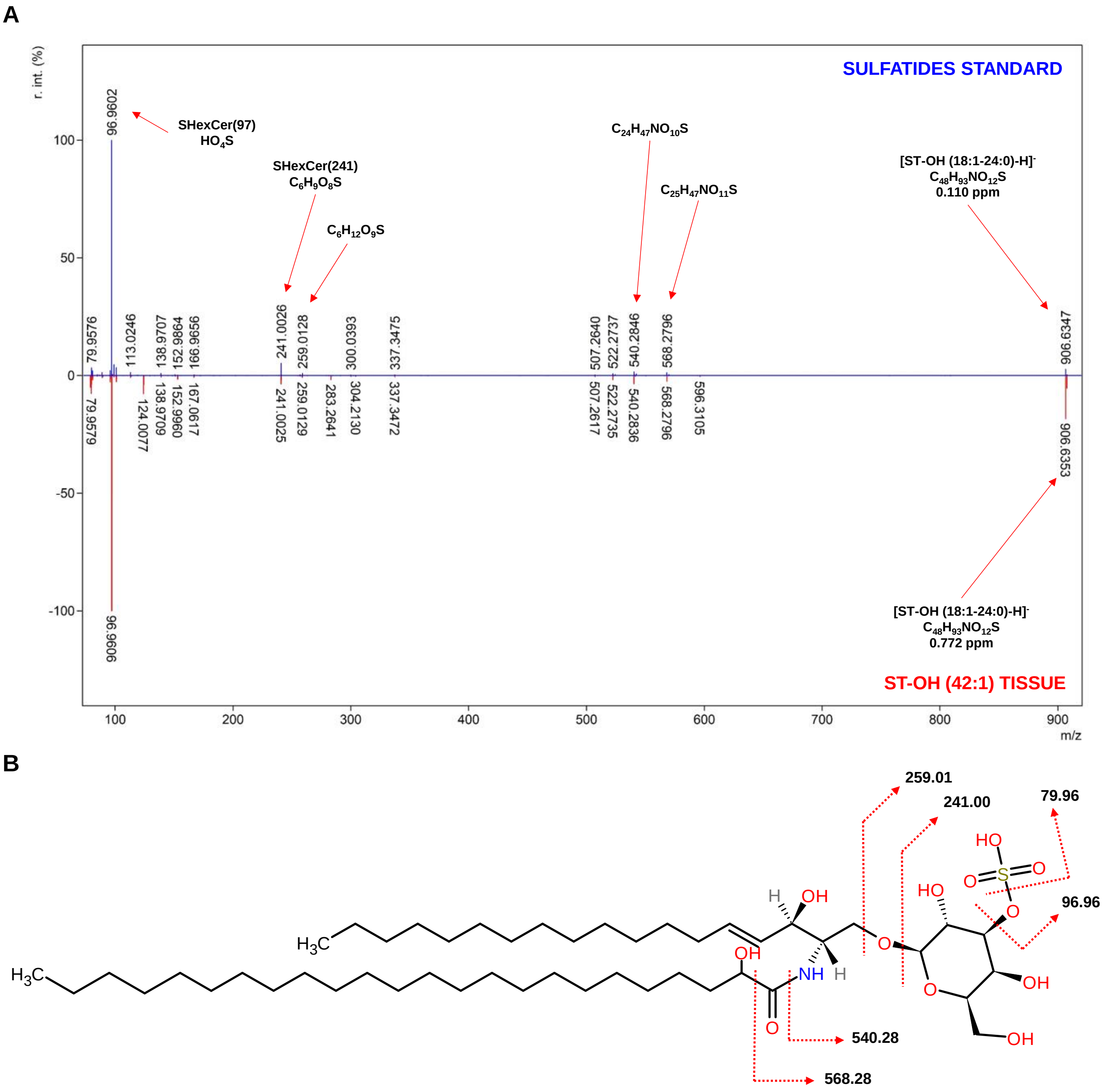

Figure S11. MALDI-MS/MS of ST-OH (18:1_24:0) in negative ion mode. A) MALDI-MS/MS of the standard mixture compared to that obtained from the liver tissue sections. B) Structure of ST-OH (18:1_24:0) annotated with the locations of fragmentation.

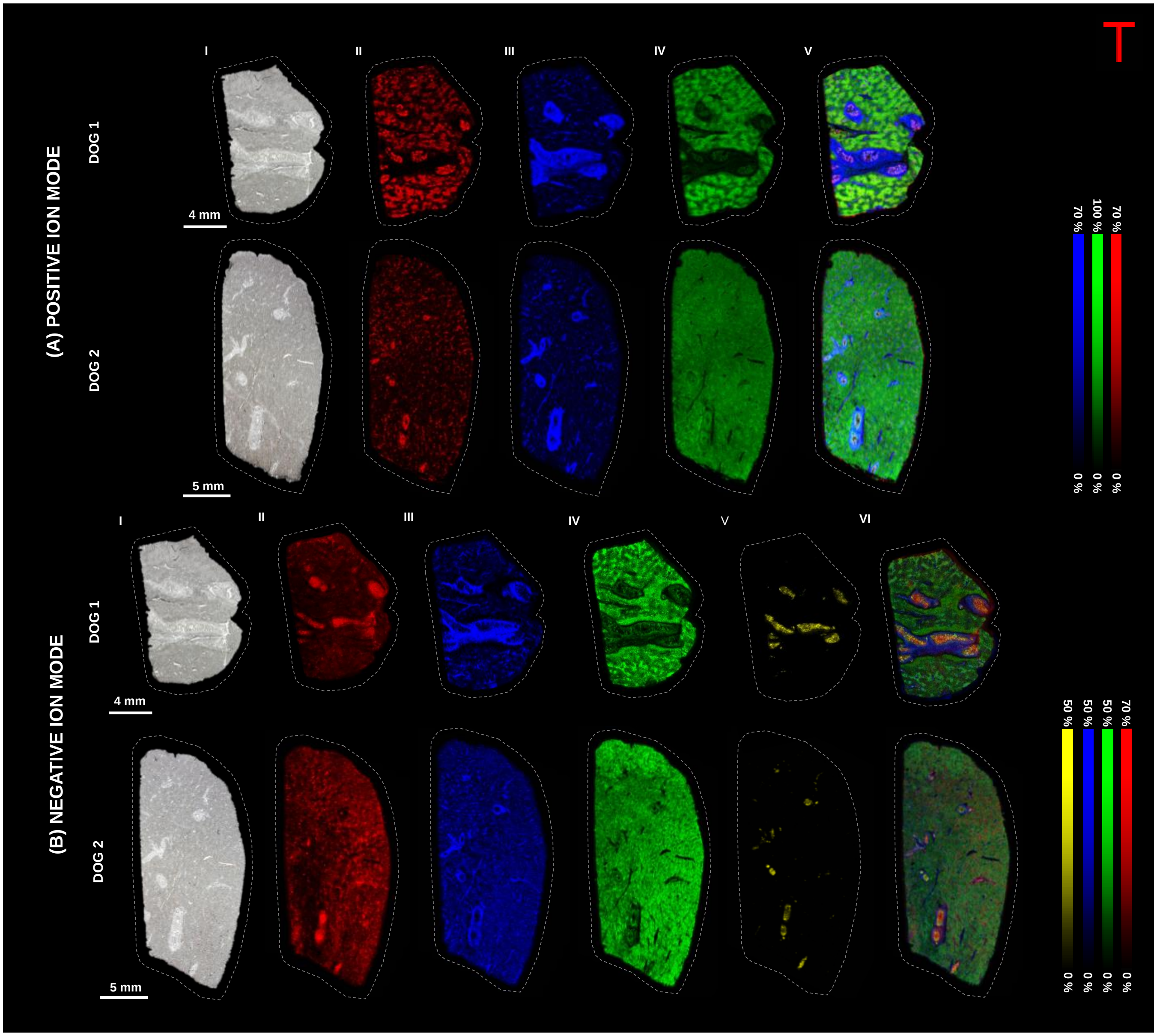

Figure S12: MALDI-MS imaging of compound A-dosed liver tissue (dogs 1 and 2). A) MALDI-MS imaging in positive ion mode showing I) optical image of tissue section before matrix application and resulting images showing the distribution of II) compound A ($[M+H]^+$) at *m/z* 502.15, III) ($[PC (32:0)+K]^+$) at *m/z* 772.52, IV) ($[PC (38:4)+K]^+$) at *m/z* 848.54 and V) overlay of selected species. B) MALDI-MS imaging in negative ion mode showing I) optical image of tissue section before matrix application and resulting images showing the distribution of II) taurocholic acid ($[M-H]^-$) at *m/z* 514.28, III) ($[SM (34:1) – CH_3]^-$) at *m/z* 687.54, IV) ($[PI (18:0\_20:4) - H]^-$) at *m/z* 885.55, V) ($[ST\text{-}OH (18:1\_24:0) - H]^-$) at *m/z* 906.64 and VI) overlay of selected species (spatial resolution 50 × 50 µm, normalized with TIC).

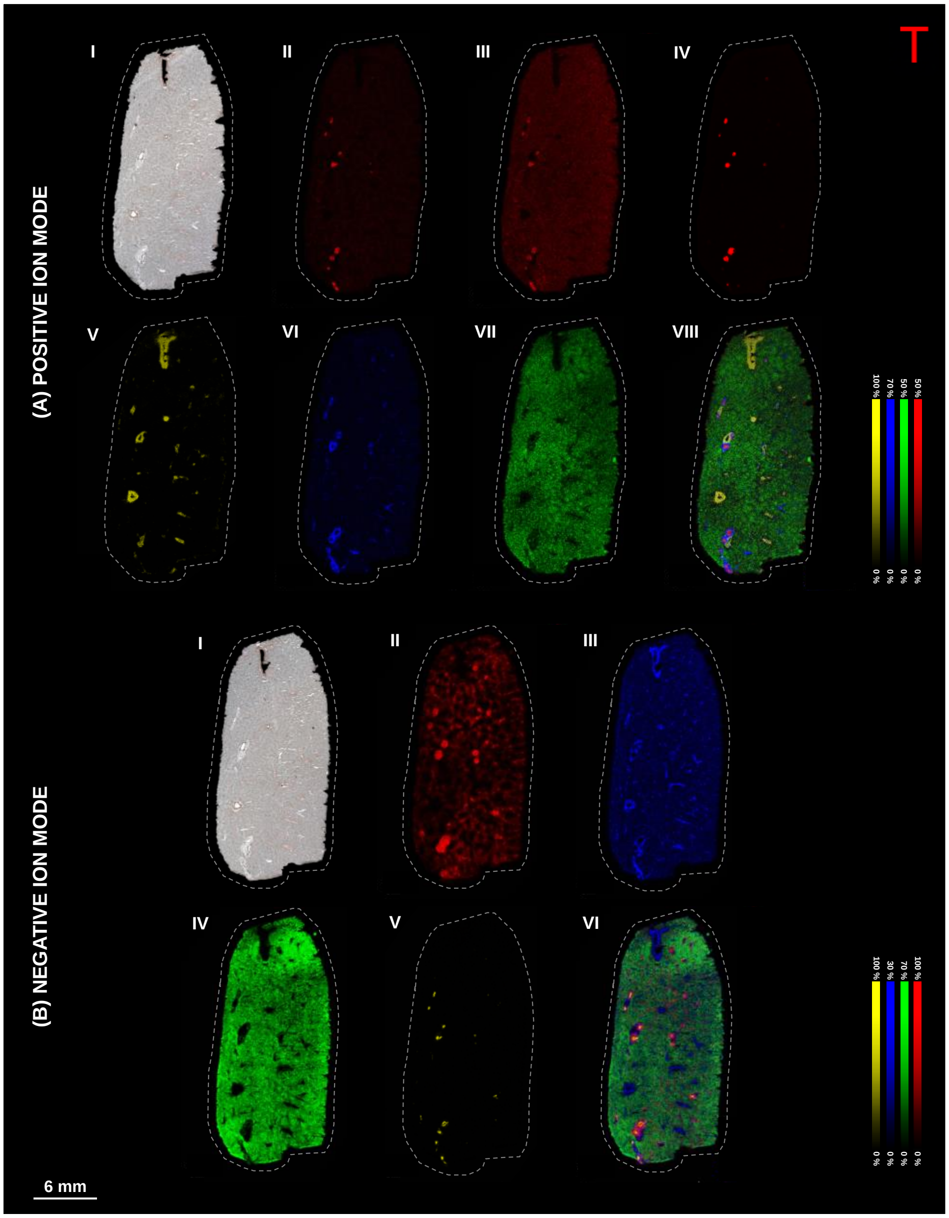

Figure S13: MALDI-MS imaging of compound B-dosed liver tissue (dog 2). A) MALDI-MS imaging in positive ion mode showing I) optical image of tissue section before matrix application and resulting images showing the distribution of II) desmethyl metabolite ($[M+H]^+$) at *m/z* 502.15, III) compound B ($[M+H]^+$) at *m/z* 516.16, IV) *N*-oxide metabolite ($[M+H]^+$) at *m/z* 532.16, V) heme ($[M]^+$) at *m/z* 616.17, VI) ($[PC (32:0)+K]^+$) at *m/z* 772.52, VII) ($[PC (38:4)+K]^+$) at *m/z* 848.54 and VIII) overlay of selected species. B) MALDI-MS imaging in negative ion mode showing I) optical image of tissue section before matrix application and resulting images showing the distribution of II) taurocholic acid ($[M-H]^-$) at *m/z* 514.28, III) ($[SM (34:1) – CH_3]^-$) at *m/z* 687.54, IV) ($[PI (18:0\_20:4) - H]^-$) at *m/z* 885.55, V) ($[ST\text{-}OH (18:1\_24:0) - H]^-$) at *m/z* 906.64 and VI) overlay of selected species (spatial resolution 50 × 50 µm, normalized with TIC).

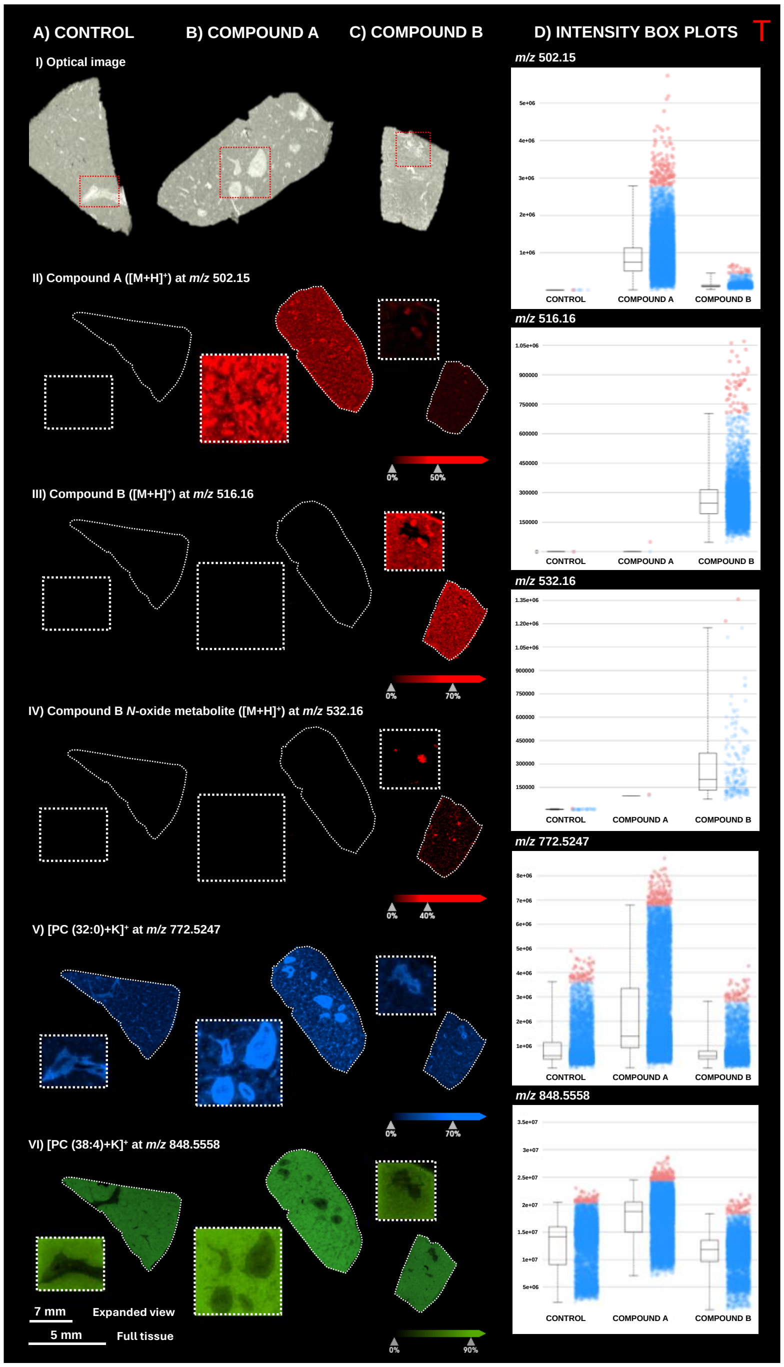

Figure S14. Comparison of A) healthy negative control, B) compound A-dosed liver, C) compound B-dosed liver, and D) intensity box plots of selected masses. I) Optical images of tissue sections before matrix applications and MALDI-MS images showing the distribution of II) compound A ($[M+H]^+$) at *m/z* 502.15, III) compound B ($[M+H]^+$) at *m/z* 516.16, IV) compound B N-oxide metabolite ($[M+H]^+$) at *m/z* 532.16, V) connective tissue marker ($[PC (32:0)+K]^+$) at *m/z* 772.5247, VI) parenchyma marker ($[PC (38:4)+K]^+$) at *m/z* 848.5568 (spatial resolution 50 × 50 µm, normalized by TIC).

A

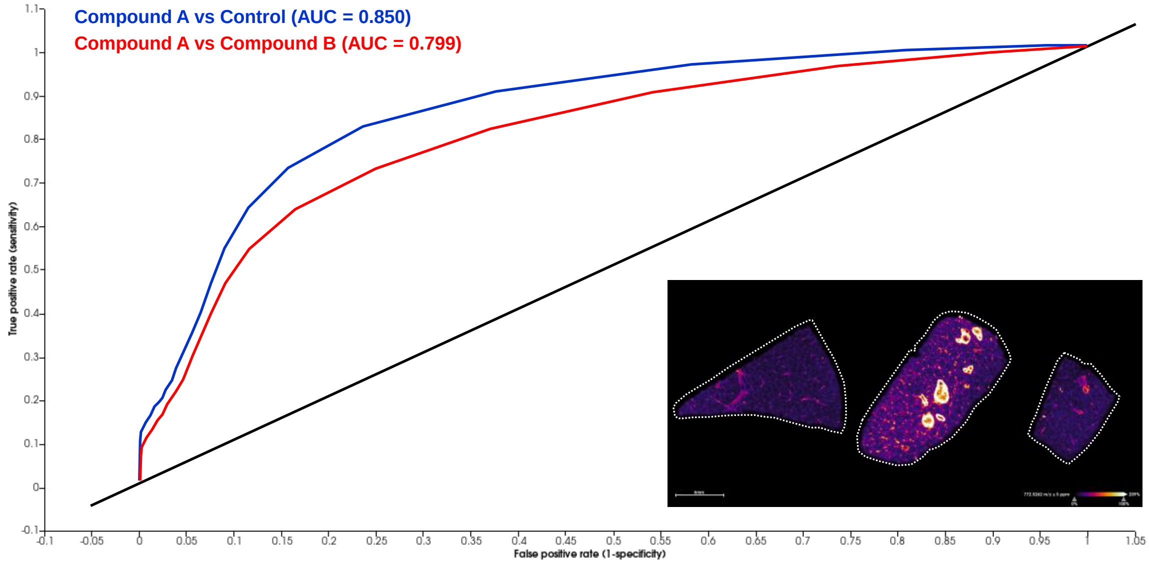

B

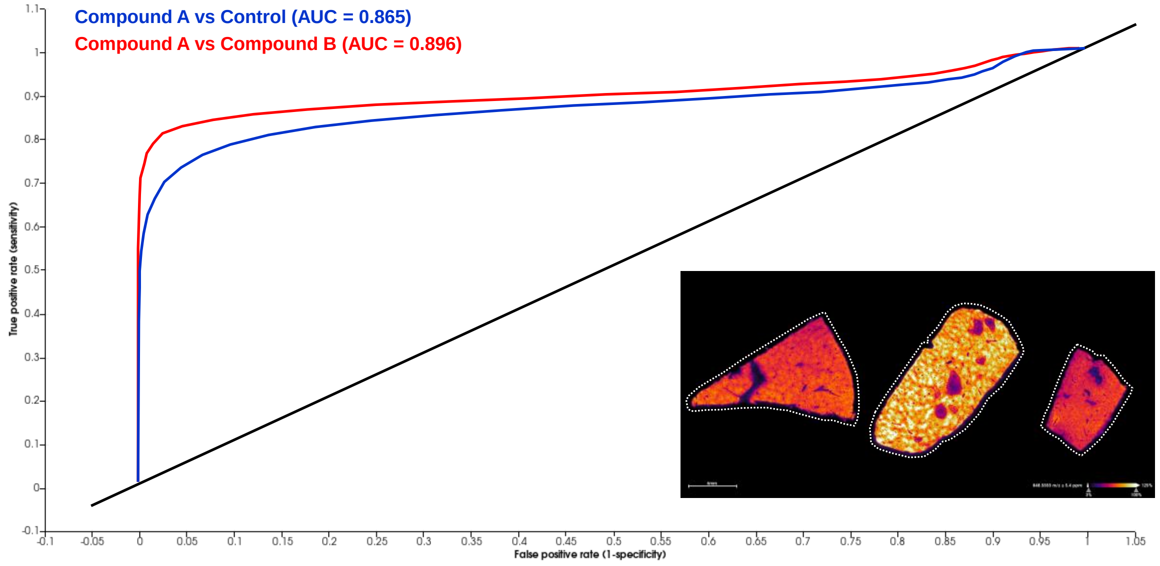

Figure S15. ROC curve comparison of compound A-dosed tissue with the control and compound B-dosed tissue. A) Connective tissue molecular marker ($[PC (32:0)+K]^+$) at *m/z* 772.5247 and B) Parenchyma molecular marker ($[PC (38:4)+K]^+$) at *m/z* 848.5568. The blue line represents the comparison between compound A dosed tissue and the control tissue. The red line represents the comparison between compound A and compound B dosed tissues (the AUC values are shown in the labels). For presentation purposes, the graphs were made by tracing the lines from the raw ROC curves and creating an overlay. Inserts show the MALDI-MS images of the selected lipid markers.
